# Preclinical safety studies of human embryonic stem cell‐derived retinal pigment epithelial cells for the treatment of age‐related macular degeneration

**DOI:** 10.1002/sctm.19-0396

**Published:** 2020-04-22

**Authors:** Sandra Petrus‐Reurer, Pankaj Kumar, Sara Padrell Sánchez, Monica Aronsson, Helder André, Hammurabi Bartuma, Alvaro Plaza Reyes, Emeline F. Nandrot, Anders Kvanta, Fredrik Lanner

**Affiliations:** ^1^ Department of Clinical Science, Intervention and Technology Karolinska Institutet Solna Sweden; ^2^ Division of Obstetrics and Gynecology Karolinska Universitetssjukhuset Stockholm Sweden; ^3^ Ming Wai Lau Center for Reparative Medicine Karolinska Institutet Stockholm Sweden; ^4^ Department of Clinical Neuroscience, Division of Eye and Vision St. Erik Eye Hospital, Karolinska Institutet Stockholm Sweden; ^5^ Sorbonne Université, INSERM, CNRS, Institut de la Vision Paris France

**Keywords:** age‐related macular degeneration, biodistribution, cellular therapy, chemically defined, human embryonic stem cells, retinal pigment epithelium, safety studies, subretinal injection, tumorigenicity, whole genome sequencing, xeno‐free

## Abstract

As pluripotent stem cell (PSC)‐based reparative cell therapies are reaching the bedside, there is a growing need for the standardization of studies concerning safety of the derived products. Clinical trials using these promising strategies are in development, and treatment for age‐related macular degeneration is one of the first that has reached patients. We have previously established a xeno‐free and defined differentiation protocol to generate functional human embryonic stem cells (hESCs)‐derived retinal pigment epithelial (RPE) cells. In this study, we perform preclinical safety studies including karyotype and whole‐genome sequencing (WGS) to assess genome stability, single‐cell RNA sequencing to ensure cell purity, and biodistribution and tumorigenicity analysis to rule out potential migratory or tumorigenic properties of these cells. WGS analysis illustrates that existing germline variants load is higher than the introduced variants acquired through in vitro culture or differentiation, and enforces the importance to examine the genome integrity at a deeper level than just karyotype. Altogether, we provide a strategy for preclinical evaluation of PSC‐based therapies and the data support safety of the hESC‐RPE cells generated through our in vitro differentiation methodology.

## Graphical abstract

Retinal pigment epithelial cells derived from human embryonic stem cells are a promising source for replacement therapy in age‐related macular degeneration. However, preclinical studies concerning safety of the derived products are indispensable. Here, we evaluate safety invitro and invivo, reassuring stability, purity, and lack of tumorigenicity or migratory potential of the derived product for safe clinical translation.

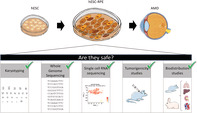

**Significance statement**

This study evaluated the preclinical safety of in vitro differentiated retinal pigment epithelial cells from embryonic stem cells by (a) examining karyotype and performing whole genome sequencing to assess genome stability; (b) performing single‐cell RNA sequencing to ensure purity and absence of undifferentiated cells; and (c) examining biodistribution and tumorigenicity of transplanted cells to rule out malignant growth and migratory properties. The derived cells proved to be safe, and this study altogether provided a strategy for preclinical evaluation of PSC‐based therapies. Also, the whole genome sequencing analysis illustrates that the preexisting load of germline variants is significantly higher than the introduced variants acquired through vitro culture or differentiation, which enforces the importance to evaluate the genome integrity at a deeper level than just karyotype.

## 1 INTRODUCTION

Age‐related macular degeneration (AMD) is the leading cause of blindness in people aged over 65 years in industrialized countries,^1^ and it manifests as dry (nonexudative) and wet (exudative) forms. The dry advanced form of the disease, also known as geographic atrophy, is untreatable at present. Although its cause is known to be multifactorial, including both genetic and environmental factors,^2^ a main hallmark is the degeneration of retinal pigment epithelium (RPE) cells: a monolayer of polarized hexagonal and heavily pigmented cells that constitutes the outer blood‐retina‐barrier and performs a number of central tasks in the eye.^3^ RPE cells are crucial for the maintenance of the photoreceptor layer, and their scarcity leads to a progressive outer retina degeneration and vision loss.^4^, ^5^, ^6^ Therefore, a potential treatment strategy would involve cell replacement of RPE cells from human pluripotent stem cells (hPSC). Several groups, including ours, have established defined, xeno‐free, and robust differentiation protocols to derive RPE cells from human embryonic stem cells (hESCs) with morphological, physiological, and functional features shared with native RPE cells.^7^, ^8^, ^9^, ^10^

Preclinical studies have shown that subretinal suspension or sheet injections of hPSC‐RPE can prevent photoreceptor and vision loss,^7^, ^11^, ^12^, ^13^, ^14^ altogether supporting its translation into the first clinical studies.^15^, ^16^, ^17^, ^18^, ^19^ In addition to cell therapies for AMD, differentiated RPEs have been used to treat patients with Stargardt's macular dystrophy.^20^ Also, other cell types derived from PSCs are currently being tested in clinical studies for the replacement of cells in cardiac ischemia, type I diabetes (trial ClinicalTrials.gov number NCT03162926 and NCT03163511) or Parkinson's disease.^21^, ^22^, ^23^, ^24^ Any cell transplantation strategy should restore function but must also be thoroughly evaluated for safety, which is of particular importance when the starting material relies on PSCs that have inherent proliferative and potential tumorigenic properties. Although guidelines for assessing safety of hPSCs are still in development, some groups have paved the path, either by reporting preclinical safety studies for their ongoing clinical trials for AMD or by suggesting possible assays for such evaluation.^16^, ^25^, ^26^, ^27^, ^28^, ^29^, ^30^

In the present study, we have performed extensive tumorigenicity and migratory tests among other whole genome‐wide studies to assess the stability and safety of our hESC‐derived RPE cells, and provide novel insights for the development of hPSC‐derived therapies.

## 2 MATERIALS AND METHODS

### 2.1 hESC‐RPE in vitro differentiation

hESC line HS980 was characterized and cultured under xeno‐free and defined conditions according to the previously described method^31^ (with ethical permit from the Swedish Ethical Review Authority, EPN 2011/745:31/3). Donors gave their informed consent for the derivation and subsequent use of the hESC lines. hESCs were cultured to confluence on 10 μg/mL recombinant human laminin (rhLN)‐521 (Biolamina) and manually scraped to produce embryoid bodies (EBs) using a 1000‐μL pipette tip as described previously in our group.^7^ EBs were cultured in suspension in low attachment plates (Corning) at a density of 5 to 7 × 10^4^ cells/cm^2^. Differentiation was performed in custom‐made NutriStem hESC XF medium without bFGF and TGFβ (Biological Industries), and 10‐μM Rho‐kinase inhibitor (Millipore, Y‐27632) was added to the suspension cultures only during the first 24 hours. Media was changed twice per week. Following 5‐weeks of differentiation, optical vesicles (OVs) were mechanically dissected out of the EBs using two needles. Cells were dissociated using TrypLE Select (ThermoFisher Scientific), followed by flushing through a 20G needle. Cells were seeded through a cell strainer (ø 40 μm, BD Bioscience) onto 20 μg/mL LN‐coated dishes at a cell density of 0.6 to 1.2 × 10^4^ cells/cm^2^ and fed twice a week with NutriStem hESC XF medium without bFGF and TGFβ (Biological Industries), resulting in a pure and homogenous hESC‐RPE culture, as described previously.^7^

### 2.2 Quantitative real‐time PCR

Total RNA was isolated using the RNeasy Plus Mini Kit and treated with RNase‐free DNase (both from Qiagen). cDNA was synthesized using 1 μg of total RNA in 20 μL reaction mixture, containing random hexamers and SuperScript III reverse transcriptase (ThermoFisher Scientific), according to the manufacturer's instructions.

TaqMan real‐time PCR master mix together with TaqMan probes (ThermoFisher Scientific) for glyceraldehyde‐3‐phosphate dehydrogenase (*GAPDH*; 4333764F), nanog homeobox (*NANOG*; Hs02387400_g1), POU class 5 homeobox 1 (*POU5F1*; Hs03005111_g1), sex‐determining region Y‐box 9 protein (*SOX9*; Hs01001343_g1), paired box 6 (*PAX6*; Hs01088112_m1), bestrophin 1 (*BEST‐1*; Hs00188249_m1), RPE‐specific protein 65 kDa (*RPE65*; Hs01071462_m1), premelanosome protein (*PMEL*; Hs00173854_m1), paired box 3 (*PAX3*; Hs00240950_m1), microphthalmia‐associated transcription factor (*MITF*; Hs001117294_m1), tyrosinase (*TYR*; Hs00165976_m1), tubulin beta 3 class III (*TBB3*; Hs00801390_s1), and microtubule associated protein 2 (*MAP2*; Hs00258900_m1) were used. Samples were subjected to real‐time PCR amplification protocol on a StepOne real‐time PCR system (Applied Biosystems). Biological triplicates were performed for every condition and technical duplicates were carried out for each reaction. Results are presented as mean ± SD.

### 2.3 Flow cytometry

hPSC‐RPE were dissociated into single cells using TrypLE select. Samples were stained with FITC mouse anti‐human TRA‐1‐60 (BD Biosciences 560380, clone [TRA‐1‐60(R)], 40 μg/mL), and APC mouse anti‐human SSEA‐4 (BD Biosciences 560219, clone [MC813‐70)], 10 μg/mL) conjugated antibodies, diluted in 2% FBS and 1 mM EDTA (ThermoFisher Scientific). Cells were incubated with the conjugated antibodies on ice for 30 minutes. Fluorescence minus one controls were included for each condition to identify and gate negative and positive cells. Stained cells were analyzed using a CytoFLEX flow cytometer equipped with 488, 561, 405, and 640 nm lasers (Beckman Coulter). Analysis of the data was carried out using the FlowJo v.10 software (Tree Star).

### 2.4 Phagocytosis assay

FITC‐labeled porcine photoreceptor outer segments (POS) were isolated and kindly provided by E. F. Nandrot from Institut de la Vision, Paris.^32^ hESC‐RPE were cultured on transwell membranes (0.33 cm^2^, Corning) coated with 20 μg/mL rhLN‐521 for 5 weeks after seeding. Cells were incubated at 37°C or 4°C for 16 hours with 2.42 × 10^6^ POS/transwell diluted in Dulbecco's modified eagle medium or CO_2_‐independent media (both from ThermoFisher Scientific), respectively. After incubation, cells were quenched with trypan blue solution 0.2% (ThermoFisher Scientific) for 10 minutes at room temperature (RT), fixed with 4% methanol‐free formaldehyde (VWR) at RT for 10 minutes and permeabilized with 0.3% Triton X‐100 in 1X Dulbecco's phosphate‐buffered saline (DPBS) for 15 minutes. Rhodamine‐phalloidin staining (1:1000, Biotinum) for 20 minutes at RT was used to visualize cell boundaries. Nuclei were stained with Hoechst 33342 (1:1000, ThermoFisher Scientific) for 20 minutes at RT.

Images were acquired with a Zeiss LSM710‐NLO point scanning confocal microscope. Post‐acquisition analysis of pictures was performed using IMARIS (Bitplane) and quantification of the number of internalized POS was performed manually.

### 2.5 Immunofluorescence

Cells were fixed with 4% methanol‐free formaldehyde at RT for 20 minutes, followed by permeabilization with 0.3% Triton X‐100 (Sigma) in DPBS for 10 minutes and blocking with 4% fetal bovine serum (FBS) and 0.1% Tween‐20 (Sigma) in 1X DPBS for 1 hour. Primary antibodies against BEST‐1 (1:100, Millipore MAB5466) and cellular retinaldehyde‐binding protein (CRALBP; 1:250, Abcam ab15051, clone [B2]) were diluted to the specified concentrations in 4% FBS, 0.1% Tween‐20, 1X DPBS solution. Primary antibodies were incubated overnight at 4°C followed by a 2‐hour incubation at RT with AlexaFluor 488 donkey antimouse IgG secondary antibodies (ThermoFisher Scientific, A21202) diluted 1:1000 in 4% FBS, 0.1% Tween‐20, 1X DPBS solution. Nuclei were stained for 20 minutes at RT with Hoechst 33342 (1:1000, ThermoFisher Scientific). Images were acquired with a Zeiss LSM710‐NLO point scanning confocal microscope. Post‐acquisition analysis of the pictures was performed using the ImageJ software.

### 2.6 Histology and tissue immunostaining

Immediately after euthanasia by intravenous injection of 100 mg/kg pentobarbital (Allfatal vet. 100 mg/mL, Omnidea), the eyes were enucleated and the bleb injection area marked with green tissue marking dye (TMD) (Histolab Products). An intravitreal injection of 100 μL fixing solution (FS) consisting of 4% buffered formaldehyde (Histolab Products AB) was performed before fixation in FS for 24 to 48 hours, and embedding in paraffin. Four‐micrometer serial sections were produced through the TMD‐labeled area and every four sections were stained with hematoxylin‐eosin (HE) (VWR). Teratomas were excised, fixed with 4% methanol‐free formaldehyde (Histolab Products AB) overnight at 4°C and paraffin embedded. Four‐micrometer tissue sections were processed further for HE staining. For immunostaining, slides were deparaffinized in xylene, dehydrated in graded alcohols, and rinsed with ddH_2_O and Tris Buffered Saline (TBS, Sigma, pH 7.6). Antigen retrieval was achieved in 10 mM citrate buffer (trisodium citrate dihydrate, Sigma, pH 6.0) with 1:2000 Tween‐20 (Sigma) at 96°C for 30 minutes, followed by 30 minutes cooling at RT. Slides were washed with TBS and blocked for 30 minutes with 10% normal donkey serum (Abcam) diluted in TBS containing 5% (w/v) IgG and protease‐free bovine serum albumin (Jackson Immunoresearch) in a humidified chamber. Primary antibodies diluted in blocking buffer were incubated overnight at 4°C: human nuclear mitotic apparatus protein (NuMA) (1:200, Abcam ab84680), BEST‐1 (1:200, Millipore MAB5466), and OCT3/4 (C‐10) (1:250, Santa Cruz Biotechnology sc‐5279). Secondary antibodies (donkey antirabbit IgG [H+L] Alexa Fluor 555 A31572 and donkey antimouse IgG [H+L] Alexa Fluor 647 A31571, both from ThermoFisher Scientific) diluted 1:200 in blocking buffer were incubated 1 hour at RT. Sections were mounted with vector vectashield with DAPI mounting medium (Vector Laboratories) under a 24 × 50 mm coverslip. Images were taken with Olympus IX81 fluorescence inverted microscope or Zeiss LSM710 point scanning confocal microscope (Carl Zeiss Meditec). Postacquisition analysis of the pictures was performed using the ImageJ software.

### 2.7 TUNEL assay

Apoptotic markers were analyzed on tissue sections by a Terminal deoxynucleotidyl transferase dUTP Nick End Labeling (TUNEL) assay using the In Situ Cell Death Detection Kit (Roche, 000000011684795910). Images were taken with an Olympus IX81 inverted epifluorescence microscope. Postacquisition analysis of the pictures was performed using the ImageJ software.

### 2.8 Karyotyping

After EB dissociation, hESC‐RPE cells were plated at a density of 9000 cells/cm^2^ in two wells of a six‐well plate coated with 20 μg/mL rhLN‐521. At day 7 (when cells were still proliferative), 8 μL Karyomax colcemid (10 μg/mL, ThermoFisher Scientific, 15212‐012) were added to each well in 3 mL of fresh differentiation medium for 28 hours. Cells from each well were dissociated with 0.5 mL of TrypLE for 8 minutes at 37°C and pooled together. After centrifugation (300*g*, 10 minutes), the cell pellet was resuspended with the remaining solution after pouring off the supernatant, and 4 mL of 0.4% KCl (Sigma) in H_2_O was slowly added for 40 minutes at RT. After centrifugation (300*g*, 10 minutes), 4 mL of 3:1 methanol:acetic acid fixative (Sigma) were slowly added to the resuspended pellet. This action was repeated twice. Finally, 1 mL of the fixative mixture was slowly added to the resuspended cell pellet. Samples were analyzed at Labmedicin Skåne, Genetiska Kliniken, Skånes Universitetssjukhus in Lund, Sweden.

### 2.9 Genotyping

Genomic DNA (gDNA) was isolated using the QIAmp DNA Mini Kit (Qiagen, 51304), and 250 ng gDNA were analyzed for copy‐number variations (CNVs) with genome‐wide human single nucleotide polymorphism (SNP) array 6.0 (Affymetrix) at Bioinformatics and Expression Analysis core facility (Karolinska Institutet, Stockholm, Sweden).

### 2.10 Whole‐genome sequencing analysis

gDNA was isolated as above and 250 ng were used for sequencing with Ilumina HiSeq X, 30X coverage. Whole‐genome paired‐end DNA sequencing reads of HS980 (p22), HS980 (p38), and hESC‐RPE cells in biological triplicate experiments were aligned to the human reference genome (NCBI reference genome GRCh37 based “human_g1k_v37_decoy”) using the Burrows‐Wheeler Aligner (BWA‐0.7.8).^33^ Sequencing quality and coverage was analyzed using QualiMap (v2.2.1).^34^ Reference genome aligned BAM files was sorted and marked duplicated using Picard (v2.0.1). “GATK Best Practice” guidelines was followed to generate analysis‐ready BAM files which includes local realignments and base quality recalibration using GATK bundle “b37” files that include data sets from HapMap, Omni, Mills Indels, and 1000 Genome Indels databases. Additionally, SNPs from NCBI‐dbSNP (dbSNP‐150) were included in the analysis. Data are available at Stockholm Medical Biobank upon request through the authors.

### 2.11 Germline single nucleotide variant discovery, filtration and annotation

Analysis‐ready BAM files of HS980 (p22) (replicates 1, 2, and 3) were processed individually using GATK 3.7 HaplotypeCaller walker in genomic variant call format (gVCF) mode with default parameters. Output gVCF files of individual HS980 (p22) replicate was then used for raw germline single nucleotide variant (SNV) identification using GenotypeGVCFs walker. Furthermore, variant quality score recalibration was performed using VariantRecalibrator walker with default parameters followed by ApplyRecaliberation walker (truth sensitivity level 99.5 for SNP and 99.0 for indels) to select filter “PASS” variants separately for individual replicates. Finally, BCFtools “isec” utility was used to identify SNVs commonly present in all three replicates for further downstream analysis.

As an additional control set for analysis, publicly available preprocessed germline SNVs from 11 participants from Personal Genome Project UK were downloaded (https://www.personalgenomes.org.uk/data/) and annotated for clinical significance.

### 2.12 Mosaic variant analysis

Analysis‐ready BAM files of HS980 (p22) (replicates 1, 2, and 3), hESC‐RPE (replicates 1, 2, and 3), and HS980 (p38) (replicates 1, 2, and 3) were used to count nucleotide base composition for 263 genomic coordinates identified as candidate mosaic variants^26^ using bam‐readcount utility with minimum mapping quality ≥20. Allele frequency (AF) was calculated by dividing the altered allele count by the total allele count for each genomic position.

### 2.13 Somatic SNV calling and annotation

Somatic SNV calling was performed using GATK 3.7‐MuTect2 in a pairwise manner with default parameters. Comparisons were made between HS980 at passage 22 (HS980 p22) and hESC‐RPE (differentiated from passage 22), followed by HS980 p22 compared with passage 38 (HS980 p38) to find somatic SNV mutations. All analysis was performed in three independent replicates. dbSNP150 and COSMIC‐v83 VCF files were considered as an argument for ‐dbsnp and ‐cosmic, respectively. In addition, filter “PASS” somatic SNVs identified as a final outcome of MuTect2 pairwise analysis were merged to create a nonredundant set of somatic SNVs for HS980‐p22 vs hESC‐RPE and HS980‐p22 vs HS980‐p38.

### 2.14 CNV analysis

In the CNV discovery phase, both advanced microarray‐ and next‐generation sequencing platform‐based approaches were used to identify potential copy‐number changes during HS980 (p22) to hESC‐RPE and HS980 (p22) to HS980 (p38) differentiation processes. gDNA of all nine samples (HS980 (p22), hESC‐RPE, and HS980 (p38) in triplicate experiments) were hybridized with the genome‐wide human SNP array 6.0 (Affymetrix).

Affymetrix CEL files were imported to the PartekGenomic Suite 6.6 (Partek Inc, St. Louis, Missouri) to perform pairwise CNV analysis. Hybridization intensity signal for each hESC‐RPE and HS980 (p38) samples were compared with HS980 (p22) control samples (pairwise comparison similar to somatic SNV analysis). The genomic segmentation algorithm (with the following parameters: minimum number of probes per segment = 10, *P*‐value threshold ≤0.001, signal to noise ratio = 0.3 and diploid copy‐number range = 1.7‐2.3) was used to identify loss and gain CNV segments. Identified replicate‐wise CNV segments were merged to create nonredundant CNV segments for hESC‐RPE and HS980 (p38) samples.

Independently, reference genome aligned whole‐genome sequencing (WGS) reads (BAM) were used to identify CNVs associated with hESC‐RPE and HS980 (p38) compared with HS980 (p22) samples in a pairwise manner. The WGS pipeline of CNVkit 0.9.3 tool^35^ with default parameters in “batch” mode was used to compare individual hESC‐RPE and HS980 (p38) samples with respective HS980 (p22) control samples. Copy‐number segments were identified using the circular binary segmentation algorithm and annotated to genes using GRCh37 annotation from Ensembl‐v75. Segments with log2 ratio ≥0.3 and ≤0.3 were classified as amplifications and deletions, respectively. Furthermore, replicate‐wise copy‐number segments were merged to create nonredundant copy‐number segments for hESC‐RPE and HS980 (p38) samples. In‐house Perl scripts were used to identify overlapping copy‐number segments for hESC‐RPE and HS980 (p38) samples.

### 2.15 Clinical interpretations

ANNOVAR utility tool integrated within UCSC Galaxy was used to functionally characterize all germline and somatic SNVs. To access clinical significance, clinically annotated SNVs from ClinVar databases (release 20 171 029) (http://www.ncbi.nlm.nih.gov/clinvar/) and cancer specific coding mutations from COSMIC databases (release 83) were downloaded. Furthermore, overlapping study was performed with identified germline and somatic SNVs using BCFtools “isec” utility. Additionally, three separate lists of cancer‐driver genes were prepared which include 723 genes from the COSMIC cancer gene census,^36^ 299 genes from Bailey et al,^37^ and 242 genes from the Shibata list.^38^

### 2.16 Single‐cell RNA sequencing

For cell preparation, one 24‐well of mature hESC‐RPE cells cultured for 5 weeks after dissociation from OVs and one 24‐well of passage 14 confluent hESC (both on hrLN‐521 coated plates) were dissociated using TrypLE Select and passed through a cell strainer (ø 40 μm, BD Bioscience). Cells were further stained with live/dead marker 7‐AAD (1:200, BD 51‐68981E) and live single cells where sorted into a 384‐well plate containing 2.3 μL of lysis buffer per well using the SORP BD FACSAria Fusion instrument. hESC‐RPE were sorted in 338 wells and hESC in 46 wells; 2 wells were left empty. A validation plate with 30 wells containing hESC‐RPE (28 wells with single cells and 2 wells with 20 cells each) and 2 wells with lysis buffer only was run as control. Cells lysates were kept at 4°C during the entire process and stored at −80°C until Smart‐Seq2 sequencing^39^ was carried out by the Eukaryotic Single Cell Genomics Facility (ESCG, SciLifeLab, Stockholm, Sweden).

For sequencing analysis, single‐cell transcriptome sequencing reads were mapped to the human genome (hg19) including ERCC sequence using STAR aligner with default settings^40^ and uniquely aligned reads were retained. The number of reads for each RefSeq and Ensemble annotated genes were calculated using featureCounts.^41^ Cells were quality‐filtered based on the exclusion criterium: had total aligned reads (within transcriptomic boundaries) lesser than 10^3^ and showed expression of fewer than 2000 unique genes. Read count matrix from quality‐filtered cells was processed using R package Seurat (version 2.2.0).^42^ Gene expression measurement was performed using NormalizeData function in Seurat with scale factor 10 000 followed by log‐transformation. RunPCA, JackStraw, FindClusters, and RunTSNE functions were used to further process the data and obtain t‐SNE cluster of cells.

### 2.17 Animals

After approval by the Northern Stockholm Animal Experimental Ethics Committee (DNRN56/15), 22 New Zealand white albino rabbits (provided by the Lidköpings rabbit farm, Lidköping, Sweden), aged 5 months and weighing 3.5 to 4.0 kg, were used in this study. All experiments were conducted in accordance with the Statement for the Use of Animals in Ophthalmic and Vision Research. After approval by the Southern Stockholm Animal Experimental Ethics Committee (DNR S14/15), 90 CIEA NOG mice (provided by Taconic, Denmark) aged 4 weeks were used in this study.

### 2.18 Subretinal transplantation in rabbits

hESC‐RPE monolayers were washed with PBS, incubated with TrypLE and dissociated into single‐cell suspensions. Cells were counted in a Neubauer hemocytometer chamber using 0.4% trypan blue (ThermoFisher Scientific), centrifuged at 300*g* for 4 minutes, and the cell pellet was resuspended in freshly filter‐sterilized 1X DPBS to a final concentration of 1000 cells/μL. Each cell suspension was then aseptically aliquoted into 600 μL units and kept on ice until surgery.

Animals were anesthetized by intramuscular administration of 35 mg/kg ketamine (Ketaminol, 100 mg/mL, Intervet) and 5 mg/kg xylazine (Rompun vet., 20 mg/mL, Bayer Animal Health), and the pupils were dilated with a mix of 0.75% cyclopentolate/2.5% phenylephrine (APL). Microsurgeries were performed on both eyes using a 2‐port 25G transvitreal pars plana technique (Alcon Accurus, Alcon Nordic). The cell suspension was drawn into a 1 mL syringe connected to an extension tube and a 38G polytip cannula (MedOne Surgical Inc). Without infusion or prior vitrectomy, the cannula was inserted through the upper temporal trocar. After proper tip positioning, ascertained by a focal whitening of the retina, 50 μL of each cell suspension (equivalent to 50 000 cells) were injected slowly subretinally approximately 6 mm below the inferior margin of the optic nerve head, forming a uniform bleb that was clearly visible under the operating microscope. Care was taken to maintain the tip within the bleb during the injection to minimize reflux. After instrument removal, a light pressure was applied to the self‐sealing suture‐less sclerotomies. Local immunosuppression with 2 mg (100 μL) of intravitreal triamcinolone (Triescence, Alcon Nordic) was administered 1 week prior to the surgery, and no postsurgical antibiotics were given in accordance with the approved ethics protocol. In animals kept for long‐term evaluation, intravitreal triamcinolone was readministered every 3 months.

### 2.19 Subcutaneous transplantation in NOG mice

hESC, EBs, and hESC‐RPE monolayers were washed with PBS, incubated with TrypLE, and dissociated to single‐cell suspension. Cells were counted in the automated cell counter Moxi Z (Orflo), centrifuged, and resuspended in NutriStem hESC XF medium (hESC) or in NutriStem hESC XF medium without bFGF and TGFβ (EBs and hESC‐RPE) to a final concentration of 0.07; 0.74; 7.46; 74.62; 746.27; 7462 cells/μL (hESC) or 74 627 cells/μL (EBs and hESC‐RPE). Each cell suspension was then aseptically aliquoted into 134 μL units, mixed with 66 μL of Matrigel Matrix (Corning, 354 277) and kept on ice until transplantation.

Two hundred microliters of the Matrigel cell suspension were injected subcutaneously in the mouse necks using a 27G needle. A total of 90 NOG mice were injected, divided into 9 groups of 10 mice each (6 groups with 10; 100; 1 × 10^3^; 1 × 10^4^; 1 × 10^5^; 1 × 10^6^ hESC, 2 groups with 1 × 10^7^ of 3‐ or 5‐weeks EBs, and 1 group with 1 × 10^7^ hESC‐RPE cells; Supplemental Table S1). Teratoma growth was monitored weekly up to 4 (mice injected with hESC) or 7 (mice injected with EBs or hESC‐RPE) months. Animals were euthanized at the end point or when the teratoma reached 1 cm^3^.

### 2.20 Biodistribution analysis

For rabbits, native RPE would most likely be removed by the mechanical pressure of the injection, but not a priori with any mechanical/chemical treatment as demonstrated previously.^7^, ^14^ In any case, if integration was successful, it implies that native RPE was removed and the retinal barrier was kept intact thus avoiding immune cell infiltration. At, 1, 4, 12 weeks (2 rabbits per time‐point) and 12 months (1 rabbit), animals were euthanized by an intravenous injection of 100 mg/kg pentobarbital (Allfatal vet. 100 mg/mL, Omnidea, Stockholm, Sweden). Immediately after, organs (lung, liver, spleen, kidneys, and heart) were independently weighted and collected into a blender (Smoothieblender, Rubicson) with 5 to 10 mL 1X DPBS. After intermittent homogenization for 10 to 20 seconds, 40 μL of the mix (corresponding to a range of 53‐240 mg of tissue per organ) was placed into a 2 mL microtube with 600μL of RLT buffer (Qiagen) supplemented with 1% 2‐mercaptoethanol (Sigma), and a second round of intermittent 10 to 20 seconds homogenization with VDI12 (VWR) followed. Three aliquots of each organ per rabbit were taken as technical replicates. Care was taken to keep the samples on ice during the procedure.

For optic nerve collection, full enucleation of the rabbit eyes took place and if a residuary optic nerve was observed, it was trimmed from the eyeball (weight ranging from 10‐30 mg) and placed into a 2 mL microtube followed by intermittent 10 to 20 seconds homogenization with VDI12 (VWR) in 350 μL of RLT buffer supplemented with 1% 2‐mercaptoethanol. The two optic nerves corresponding to both eyes from the same rabbit were pooled together, when possible.

For vitreous collection, a 1000‐μL pipet tip was introduced through the optic nerve hole (after its removal) and the vitreous was sucked with the pipet and placed into a 2 mL microtube followed by intermittent 10 to 20 seconds homogenization with VDI12 (VWR) in 350 μL of RLT buffer supplemented with 1% 2‐mercaptoethanol.

For hESC or hESC‐RPE cell spiking in rabbit tissues, six serial dilutions of cells (ranging from 10 to 1 × 10^6^ cells) were made and mixed individually with 18.5 mg of tissue diluted in RLT supplemented with 1% 2‐mercaptoethanol (Figures S5A,B).

For mice, 7 months after subcutaneous injection of 10 million hESC‐RPE cells, euthanasia was performed by CO_2_ inhalation in 7 mice. Immediately after, organs (lung, liver, spleen, kidneys, heart, and gonads) and transplanted cells were independently collected into 2 mL microtubes and weighed. Twenty milligram of each organ was intermittently blended for 10 to 20 seconds with 1 mL of 1X DPBS with a VDI12 (VWR) homogenizor in 600 uL of RLT buffer supplemented with 1% 2‐mercaptoethanol. Three aliquots of each organ per mouse were taken as technical replicates into 600 μL of RLT supplemented with 1% 2‐mercaptoethanol. Care was taken to keep the samples on ice during the procedure.

For hESC or hESC‐RPE cell spiking in mouse tissues, five or seven serial dilutions of cells (ranging from 1 to 1 × 10^6^ cells) were made and mixed individually with 20 mg of tissue diluted in RLT supplemented with 1% 2‐mercaptoethanol (Figures S5C,D).

RNA isolation followed as described in the corresponding section above and quantitative real‐time PCR using the SYBR green protocol (Qiagen) and human ribosomal protein lateral stalk subunit p0 (*RPLPO*) primers (Qiagen, PPH21138F_200) was performed for all samples on a StepOne real‐time PCR System (Applied Biosystems). Biological triplicates were performed for every condition and technical duplicates were carried for each reaction.

Calculation of the equation relating log (cell/mg tissue) with threshold cycle (Ct) value allowed the inference of the amount of cells/mg present in each of the analyzed organs based on the obtained Ct values.

## 3 RESULTS

### 3.1 Efficient differentiation of functional RPE from hESC

**FIGURE 1:**
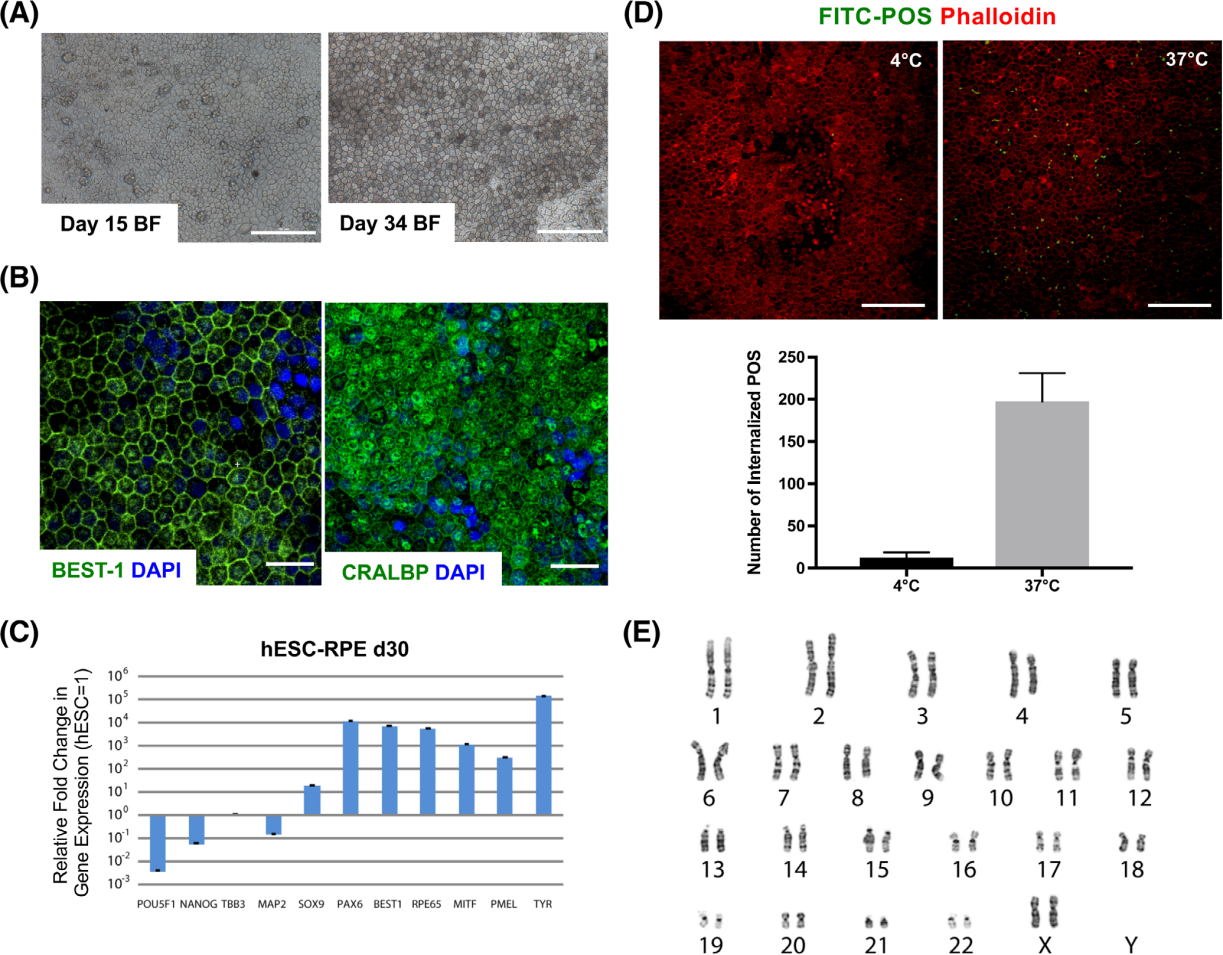
Xeno‐free and defined RPE differentiation from hESC. A, Bright‐field (BF) images of hESC‐RPE cultured on rhLN‐521 at day 15 and upon maturation at day 34. B, Immunostaining images showing Bestrophin1 (BEST‐1) and cellular retinaldehyde‐binding protein (CRALBP). C, Gene expression analysis of day 30 hESC‐RPE differentiated on hrLN521. Values are normalized to *GAPDH* and displayed as relative to undifferentiated hESC. D, Upper panel: Immunofluorescence images depicting phagocytosis of FITC‐labeled POS by day 30 hESC‐RPE cultured on hrLN‐521 after overnight incubation at 4°C (negative control) or 37°C. Lower panel: Bar graph representing the number of internalized POS in both conditions. Membrane boundaries are shown by Phalloidin staining. E, Karyotype of day 30 hESC‐RPE seeded on hrLN‐521. Bars represent mean ± SD from three independent experiments. Scale bars = 100 μm (A, D), 30 μm (B). hESC, human embryonic stem cell; POS, photoreceptor outer segment; RPE, retinal pigment epithelial

Cells were differentiated in accordance with our previously published xeno‐free and defined methodology.^7^ In brief, hESCs were aggregated as EBs in NutriStem hESC XF medium without exogenous growth factors. After 5 weeks, the pigmented structures were manually dissected and plated following single cell dissociation on (rhLN)‐521 for an additional 5 weeks to generate mature pigmented hexagonal hESC‐RPE (Figure 1A). hESC‐RPE were uniformly positive for protein expression of specific RPE markers such as BEST‐1 and CRALBP (Figure 1B). Transcriptional analysis showed robust expression of neuroectoderm transcripts *SOX9* and *PAX6*, and RPE differentiation was apparent by the expression of genes such as *BEST‐1*, *RPE65*, *MITF*, *PMEL*, and *TYR*, whereas pluripotency‐related gene expressions were low (Figure 1C). At a functional level, cultures showed active phagocytosis of isolated FITC‐labelled bovine POS at 37°C when compared to the 4°C condition (Figure 1D). Finally, hESC‐RPE showed a normal karyotype with no clonal aberrations (Figure 1E).

### 3.2 Germline variations in the starting hESC

**FIGURE 2:**
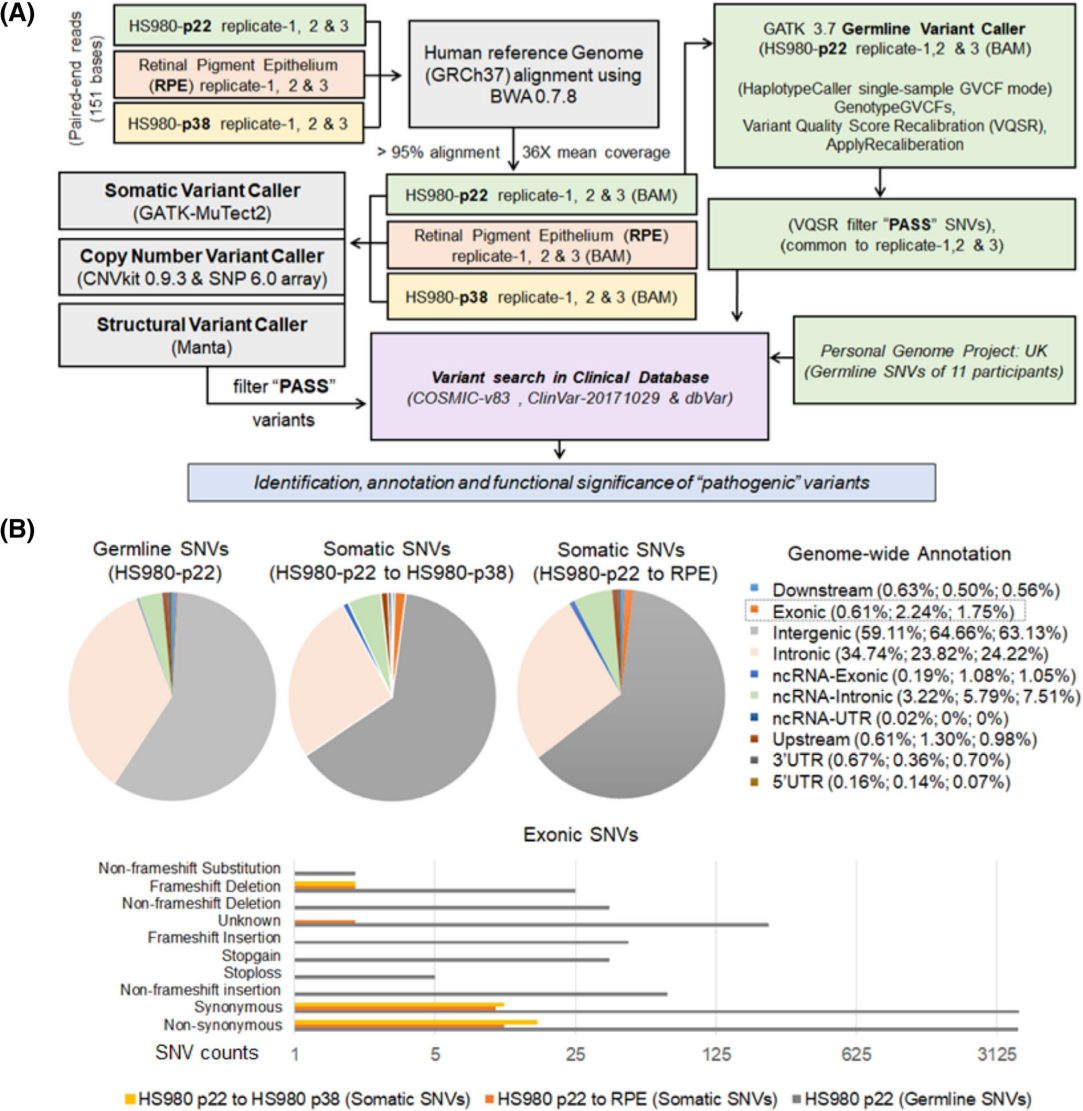
Whole‐genome sequencing of hESC and differentiated RPE cells. A, Flowchart describing HS980 (p22), hESC‐RPE, and HS980 (p38) whole‐genome DNA sequence analysis pipelines for various germline and somatic variant calling, filtration, and annotation. B, Upper panel: Genome‐wide functional annotation of germline and somatic SNVs based on their relative location in the genome. Lower panel: Bar chart showing different annotation for germline and somatic Exonic SNVs. hESC, human embryonic stem cell; RPE, retinal pigment epithelial; SNV, single nucleotide variant

Genetic stability is a very relevant concern to translate PSC‐based therapies clinically. This includes the presence of germ line mutations in the starting cell line and culture‐induced changes acquired during cell expansion and in vitro differentiation. To unambiguously detect germline mutations, we would have to analyze cells of the original embryo from which the hESC line was established from or by parallel sequencing of the couple that donated the embryo. Since this was not a possible option, in this study we performed WGS of hESCs at the initiation of differentiation at passage 22 and in the resulting hESC‐RPE. We also included hESCs cultured in parallel up to passage 38 to compare the extent of culture‐induced alterations, whereas cells are undifferentiated compared to in vitro differentiation. More than 95% of raw sequencing reads (151 paired‐end bases, mean coverage 36X) aligned to the NCBI reference genome GRCh37 based “human_g1k_v37_decoy” using BWA‐MEM (Table S2). The mapped reads were investigated further to identify putative germline and somatic SNVs, CNVs and large structural variants (SVs) following the Genome Analysis Toolkit (GATK) best practice guidelines (Figure 2A).

Relative to the reference genome, 4 355 521 (3 700 632 SNPs and 657 795 Indels) germline SNVs were identified for the starting hESC (HS980, passage 22) cells using GATK 3.7 HaplotypeCaller. Next, mutational subtype analysis of these SNPs revealed that transition (Ts) was more common than transversion (Tv) (Figure S1), and heterozygous variants contribution was more than homozygous variants (62% and 38%, respectively). Furthermore, 67.99% SNVs (2 596 074 SNPs and 365 547 Indels) were identified as common variants and 32% SNVs (1 104 558 SNPs and 292 248 Indels) were identified as noncommon variants based on their global allele frequency (≥0.1% in at least one 1000 genome population) in dbSNP 150. Additionally, genome‐wide classification of noncommon SNVs using ANNOVAR^43^ revealed their widespread presence within intergenic and intronic genomic regions, whereas only 0.61% SNVs were annotated within exonic boundaries (Figure 2B).

To characterize the clinical relevance of all identified germline SNVs (both common and noncommon), their presence in the Catalog of Somatic Mutation in Cancer (COSMIC release 83) and ClinVar (release 20 171 029) databases was analyzed. In all, 9519 and 17 265 SNVs reported in ClinVar and COSMIC (coding) databases were identified, respectively. Further investigation revealed that 20 germline SNVs were reported as “pathogenic” in ClinVar (Table S3), whereas 35, 20, and 18 germline SNVs with pathogenic FATHMM score^44^ were annotated within COSMIC cancer gene census^36^, ^45^ (Table S4A), Bailey et al, cancer‐driver genes^37^ (Table S4B), and Shibata cancer‐driver genes^38^ (Table S4C), respectively. It is important to note that all SNVs reported as pathogenic by ClinVar and 34 out of 35 localized within COSMIC cancer gene census were categorized as a common variants in dbSNP 150. In order to compare the extent of clinically relevant germline SNVs in our samples with any healthy individual, we included germline SNVs of 11 participants from the Personal Genome Project UK (https://www.personalgenomes.org.uk/). Interestingly, ClinVar reported 24 (average) SNVs as pathogenic, whereas COSMIC cancer gene census, Bailey et al, cancer‐driver genes, and Shibata cancer‐driver genes lists reported 34.09, 22.90, and 16.45 (average) SNVs as pathogenic, respectively (Table S7). Importantly, we noted that our hESC source samples (HS980, passage 22) and normal participants show comparable load of clinically relevant germline SNVs, illustrating the challenge of finding a starting material that would be free of any inherited germline variant listed in COSMIC as pathogenic.

### 3.3 Mosaic variations in hESC and differentiated hESC‐RPE cells

We next compared mosaic variant (SNVs) status in our starting hESC (HS980, passage 22), the final hESC‐RPE product and cultured hESC (HS980, passage 38) cells, with 263 candidate mosaic variants identified using whole‐exome sequencing (WES) datasets of 140 independent hESC lines including 26 lines prepared for potential clinical use.^26^ AF for all 263 genomic positions was calculated using bam‐readcount utility with minimum mapping quality ≥20. Variant analysis resulted in four genomic positions (chr1: 169500014, A/G; chr3: 49713924, C/G; chr5: 52382888, C/G; and chr11: 128773338, C/G) with heterozygous AF (AF ≥0.35 and AF ≤0.75) common to all samples and replicates. Further analysis with common altered allele (AF ≥0.05 in any two out of three replicates) resulted in 3, 1, and 2 mosaic variants for HS980 (p22), hESC‐RPE, and HS980 (p38) samples, respectively. Importantly, none of the identified mosaic variants were commonly present within the samples neither within *TP53*, as reported earlier.^26^

### 3.4 Acquired SNV changes during hESC culture and hESC‐RPE differentiation

We next explored the existence of acquired somatic mutations during differentiation that could eventually be detected in the final hESC‐RPE product. Therefore, we investigated how many mutations occur during undifferentiated culture for a similar time period as the differentiation by sequencing the genome of hESCs at passage 38. Somatic variant caller MuTect2^46^ from the Genome Analysis Toolkit (GATK 3.7) was applied in a pairwise analysis mode to identify 1543 and 1496 nonredundant sets of filter “PASS” somatic SNVs for hESC‐RPE and HS980 (p38) samples, respectively, after merging replicate‐wise SNVs. Subsequently, their genome‐wide annotations and clinical relevance were evaluated with similar approaches used for germline SNVs. Interestingly, 25 and 32 somatic SNVs were observed within exonic and the splicing boundaries for hESC‐RPE and HS980 (p38) samples, respectively (Tables S5 and S6). Single‐cell gene expression analysis revealed that majority of genes with nonsynonymous exonic changes were not expressed in either hESC or hESC‐RPE cells except for ZFYVE16, FAM8A1, METTL9, and ZXDB that showed expression in hESC (HS980 (p38)) or hESC‐RPE samples (Tables S5 and S6).

Furthermore, clinical significance analysis of all somatic SNVs resulted in eight SNVs overlapping with COSMIC database for both hESC‐RPE and HS980 (p38) samples, whereas none of the observed somatic SNVs were reported as pathogenic in ClinVar database. Additionally, characterization of COSMIC overlapping SNVs resulted in pathogenic FATHMM score for COSM1045366 (NBEAL2) and COSM6417107 (METTL9)—for hESC‐RPE samples—and COSM711096 (FAM108A1/ABHD17A) for HS980 (p38) samples. However, none of the COSMIC overlapping SNVs were within any cancer‐driver genes.

### 3.5 Somatic CNVs during hESC‐RPE differentiation

Both array‐based genomic hybridization and next‐generation WGS‐based methods were used to identify any possible copy‐number changes in hESC‐RPE and HS980 (p38) cells during the differentiation process. Analysis of whole‐genome DNA sequencing datasets using CNVkit 0.9.3 tool resulted in 291 (98 amplifications and 193 deletions) and 283 (88 amplifications and 195 deletions) nonredundant sets of somatic CNVs for hESC‐RPE and HS980 (p38) samples, respectively. Further investigation revealed copy‐number changes for 499 and 461 nonredundant genes (UCSC hg19 gene annotation) in hESC‐RPE and HS980 (p38) cells, respectively. To examine their clinical relevance, the presence of amplification or deletion associated genes were searched in three cancer‐driver gene lists. Four cancer‐driver genes *NOTCH2*, *ASXL1*, *PDE4DIP*, and *MUC4* were found to be selectively amplified in both hESC‐RPE (Figure S2A) and HS980 (p38) (Figure S2B) samples. Further investigation revealed that they are somatic mutational changes, not amplified or deleted in COSMIC Cancer Gene census (CGC). Moreover, overlapping status of copy‐number changes identified for hESC‐RPE and HS980 (p38) samples were checked, finding that more than 65% of amplification and 75% of deletion events are shared in these two samples, therefore suggesting that these copy‐number changes are a consequence of time in culture rather than due to the differentiation protocol.

From the array data, 625 (140 amplifications and 485 deletions) and 590 (138 amplifications and 452 deletions) nonredundant sets of somatic copy number changes were identified for hESC‐RPE and HS980 (p38) samples, respectively, using PartekGenomic Suite 6.6 (Partek Inc). For clinical significance, a similar analysis was performed by evaluating copy number's gain or loss status in COSMIC CGC database. Importantly, none of the copy number changes were found to be reported in COSMIC.

### 3.6 Structural changes during hESC‐RPE differentiation

WGS datasets were used to identify putative large‐scale SVs in hESC‐RPE and HS980 (p38) cells using Manta^47^ in a pairwise analysis mode. This analysis resulted in somatic translocation break ends (BND), inversion (INV), insertion (INS), segmental deletion (DEL), and tandem duplications (DUP: TANDEM). 21 (15 BND, 3 DEL, 2 INV, and 1 DUP: TANDEM) and 19 (12 BND, 3 DEL, 2 INV, and 1 DUP: TANDEM) nonredundant somatic SVs were identified for hESC‐RPE and HS980 (p38) samples, respectively. Further clinical relevance analysis did not result in any SV affecting cancer‐driver genes.

### 3.7 Differentiation protocol generates highly pure hESC‐RPE cells

**FIGURE 3:**
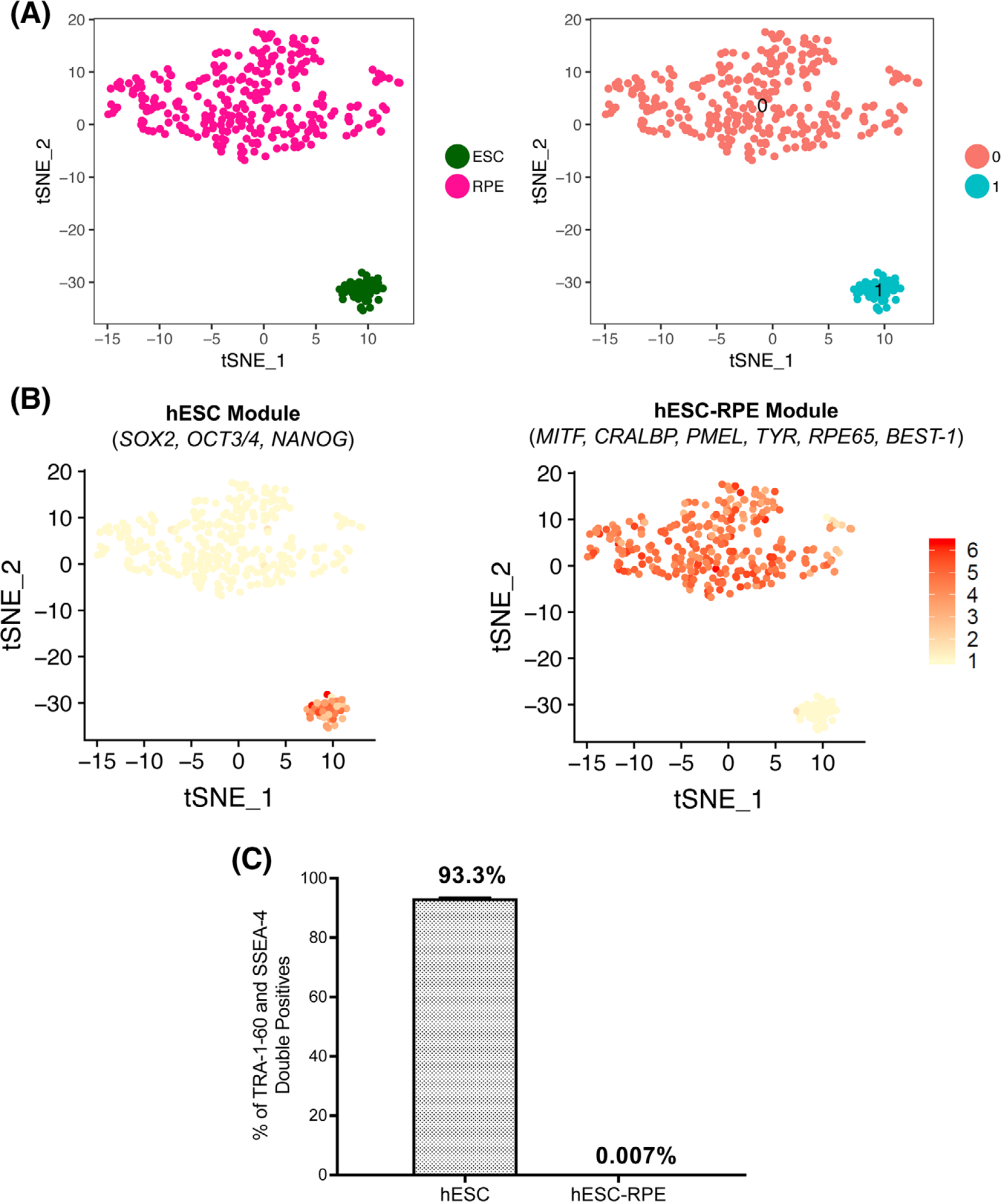
Cell purity assessment of hESC‐RPE. A, Left panel: t‐SNE distribution of 42 hESC and 285 differentiated hESC‐RPE cells. Right panel: Unbiased clustering of analyzed cells based on differentially expressed genes. B, Modular expression of selective hESC and hESC‐RPE markers in hESC and hESC‐RPE cluster of cells. C, Bar graph showing the percentage of hESC or hESC‐RPE co‐expressing SSEA‐4 and TRA‐1‐60. Bars represent mean ± SD from three independent experiments. hESC, human embryonic stem cell; RPE, retinal pigment epithelial

Another specific concern with PSC‐based cell therapies is the risk of having remaining undifferentiated cells in the final product, which could proliferate and generate teratomas. qPCR analysis showed strong reduction of pluripotency markers *NANOG* and *POU5F1* (Figure 1C), suggesting removal of undifferentiated cells with the hESC‐RPE differentiation protocol. However, there could still remain low levels of undifferentiated cells, partially differentiated cells, and cells of alternative cell lineages. To unbiasedly identify any potential remaining undifferentiated hESC or contamination of alternative cell types in the differentiated hESC‐RPE cells, Smart‐Seq2 protocol was performed based on single‐cell transcriptome sequencing of undifferentiated hESCs and differentiated hESC‐RPE cells. After reference genome (hg19) alignment and quality control, 327 high‐quality single‐cell transcriptomes (285 hESC‐RPE and 42 hESC) were retained with an average of 10^4^ sequences depth per cell. As a result of analysis, two distinct clusters of cells representing hESC and hESC‐RPE cells were identified (Figure 3A). The expression dynamics of selective marker genes in these two clusters further highlighted complete differentiation of hESC into hESC‐RPE cells with no remaining undifferentiated cells in the hESC‐RPE samples at a transcriptomic level. Module expression for hESC marker genes (*SOX2*, *POU5F1*, and *NANOG*) was exclusively observed in the cluster containing hESC cells whereas module expression for RPE marker genes (*MITF*, *CRALBP*, *PMEL*, *TYR*, *RPE65*, and *BEST‐1*) in the cluster containing hESC‐RPE cells (Figure 3B). Additionally, high expression of *PMEL* and *BEST‐1* was noted in the majority of hESC‐RPE cluster compared to hESC cluster (Figure S3). Importantly, all 285 hESC‐RPE cells expressed several RPE marker genes, indicating that the cultures are highly pure. A limitation of the scRNAseq analysis performed in this study is the relative low number of cells analyzed. We therefore performed flow cytometry in three independent samples of a million cells each showing 0.007% of TRA‐1‐60 and SSEA‐4 double positive cells in the final hESC‐RPE product (Figure 3C).

### 3.8 Nontumorigenic growth after hESC‐RPE injection

**FIGURE 4:**
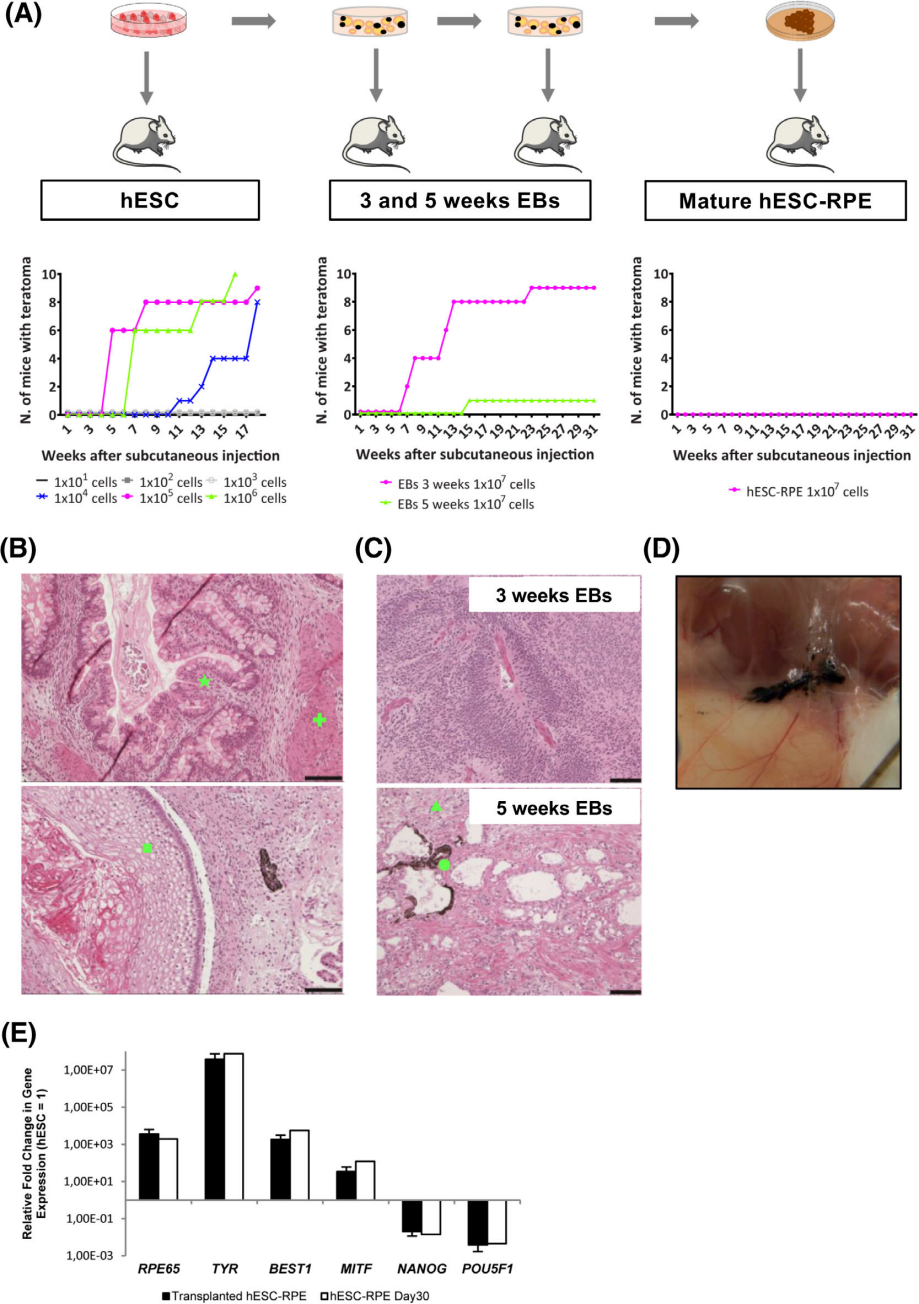
Evaluation of teratoma potential of hESCs, 3‐ and 5‐week embryoid bodies and mature hESC‐RPE. A, Flowchart and graphs showing teratoma growth after injection of cells at several time points of the differentiation protocol. B, HE staining images of hESC‐derived teratomas showing representative tissues of the three germ layers: endodermal tubules with numerous Goblet cells (★), bundles of smooth muscle (**+**), and stratifying squamous epithelium (◼). C, HE staining images of teratoma sections derived from 3‐week EBs showing neuroectodermal features, and 5‐week EBs showing neuropil‐like structures (▲) and pigmented cells (●). D, Subcutaneous picture of the neck of a mouse showing pigmented hESC‐RPE 7 months after injection. E, Gene expression analysis of day 30 hESC‐RPE 7 months after subcutaneous injection. Values are normalized to *GAPDH* and displayed as relative to undifferentiated hESC. Bars represent mean ± SD from three independent experiments. Scale bars = 100 μm (B, C). EB, embryoid body; HE, hematoxylin‐eosin; hESC, human embryonic stem cell; RPE, retinal pigment epithelial

Although genomic and transcriptional analyses are informative, they still should be complimented by functional studies. Tumorigenicity studies were therefore performed to evaluate the risk of tumorigenic growth capacity of the hESC‐RPE cells, especially of undifferentiated pluripotent cells. A well‐established methodology, also recommended by the International Stem Cell Initiative,^48^, ^49^ is to screen for tumor formation following subcutaneous injection in immunocompromised NOG mice.^50^ A benefit with subcutaneous injection is that the cell number is not as limited as it is in the subretinal space (the planned clinical site of delivery). Six groups of NOG mice (10 mice per group) were subcutaneously injected with increasing numbers of hESC (ranging from 1 to 10 million cells) to establish the number of cells that could potentially generate a tumorigenic growth. In parallel, 10 million cells from three time points along the differentiation protocol were injected in three groups (Table S1). 10/10, 9/10, and 8/10 mice injected with 1 million; 100 000 and 10 000 hESCs, respectively, developed detectable cell masses, which were not observed in any of the mice injected with lower amounts of hESCs. All teratomas analyzed showed contribution to all three germ layers (Figure 4B). A subset showed formation of yolk sac tissue that has been suggested to be a malignant indicator associated with teratocarcinoma (Figure S4A). Furthermore, 9/10 and 1/10 mice injected with 10 million cells obtained from partially differentiated EBs that were in culture for 3 and 5 weeks respectively showed cell mass formation (Figure 4A). Importantly, and in contrast to the hESC‐derived teratomas, these masses were only comprised of tissue with neuroectodermal features (Figure 4C), therefore indicating that cells at 3 and 5 weeks have the capacity to exit the pluripotent state and differentiate into neurectoderm despite still being proliferative. Most importantly, 7 months after hESC‐RPE injections, no growth could be detected and the remaining cells were pigmented and expressed RPE‐specific markers. In addition, they also appeared viable without signs of extensive apoptosis and localized only at the injection site (Figures 4D,E; Figure S4B). Several studies have also examined the tumorigenic risk at the clinical site (Table 1).^10^, ^15^, ^17^, ^27^, ^28^, ^30^, ^51^ Analysis of 26 rabbit eyes transplanted with the clinical dose subretinally (50 000 cells in 50uL) with confirmed integration (evaluated by pigmentation) and no obvious signs of rejection at 4 weeks showed no signs of tumor formation or growth. Furthermore, histology in integrated hESC‐RPE cells at 1, 3, and 8 months after transplantation lacked the expression of OCT3/4 thus confirming the absence of undifferentiated cells after transplantation (Figures S4C,D).

**TABLE 1:** Summary of the current clinical trials for AMD and Stargardt's disease linked to the methodologies used in their preclinical studies

Study	Cells/disease	Trial/clinical phase	Karyotyping/sequencing	Biodistribution/migration	Teratoma	Preclinical models
Schwartz et al^17^ Song et al^15^ Lu et al^28^	hESC‐RPE suspensions for dry AMD and Stargardt's macular distrophy (18 patients)	Astellas Institute for Regenerative Medicine, Massachusetts, USA (NCT01344993). Phase I completed	G‐band karyotyping; global gene expression analysis	—	(NIH)‐III immune‐deficient mice (subretinal space, 6 per cohort) at 1, 3, and 9 months; in 79 immunosuppressed RCS rats at 24 weeks (subretinal space; 1e5 hESC‐RPE cells)	Rodent
Mandai et al^16^	hiPSC‐RPE patch on a collagen gel for wet AMD (6 patients)	RIKEN Center for Developmental Biology, Kobe, Japan (NCT01691261). Phase I interrupted	G‐band karyotyping; whole genome/exome sequencing (WGS/WES); SNP‐genotyping array genomic; RNA‐sequencing; DNA methylation analysis; single‐cell gene expression analysis	—	In 5 NOG immune‐deficient mice at 8 and 24 weeks and lifelong (subcutaneously; 1e6 hiPSC‐RPE cells)	Rodent
Kashani et al^19^ Koss et al^29^ Diniz et al^30^	hESC‐RPE patch on parylene scaffold for dry AMD (20 patients)	California Project to Cure Blindness/Regenerative Patch Technologies, Ltd, California, USA (NCT02590692). Phase I/II completed	—	In 69 nude rats at 1, 6, and 12 months by HE and immunofluorescence staining (retina and optic disc)	In 69 nude rats at 1, 6, and 12 months (subretinal space; 1e5 hESC‐RPE cells or 0.4 mm^2^ hESC‐RPE patch); and in 14 immunosuppressed Yucatán minipigs, 4 weeks (subretinal space; 1e5 hESC‐RPE cells)	Rodent and Yucatán minipigs
Da Cruz et al^15^	hESC‐RPE patch on a human‐vitronectin‐coated polyester membrane for acute wet AMD (10 patients)	London Project to Cure Blindness/University College London, UK (NCT01691261). Phase I completed	G‐band karyotyping	In 20 pigs at 26 weeks by qPCR (sites: adrenal, bone marrow [rib and femur], brain, heart, kidneys, liver, lungs, lymph nodes, optic nerve, spleen and thymus)	In 80 (NIH)‐III immune‐deficient mice at 26 weeks (subretinally, intramuscularly and subcutaneously; 6e4 hESC‐RPE cells)	Rodent and pig
Sharma et al^27^	hiPSC‐RPE patch on PLGA scaffold	—	G‐band karyotyping and sequencing of coding regions of 223 onco‐genes across nine iPSC clones	—	In immunocompromised rats (Crl:NIH‐Foxn1rnu, 15 per cohort) at 5‐7 weeks (subretinal space; 1e5 hiPSC‐RPE cells or 0.5 mm‐diameter hiPSC‐RPE patch)	Rodent and pig

Abbreviations: AMD, age‐related macular degeneration; HE, hematoxylin‐eosin; hESC, human embryonic stem cell; RPE, retinal pigment epithelial; SNP, single nucleotide polymorphism.

### 3.9 Biodistribution studies only detect cells at site of transplantation

**FIGURE 5:**
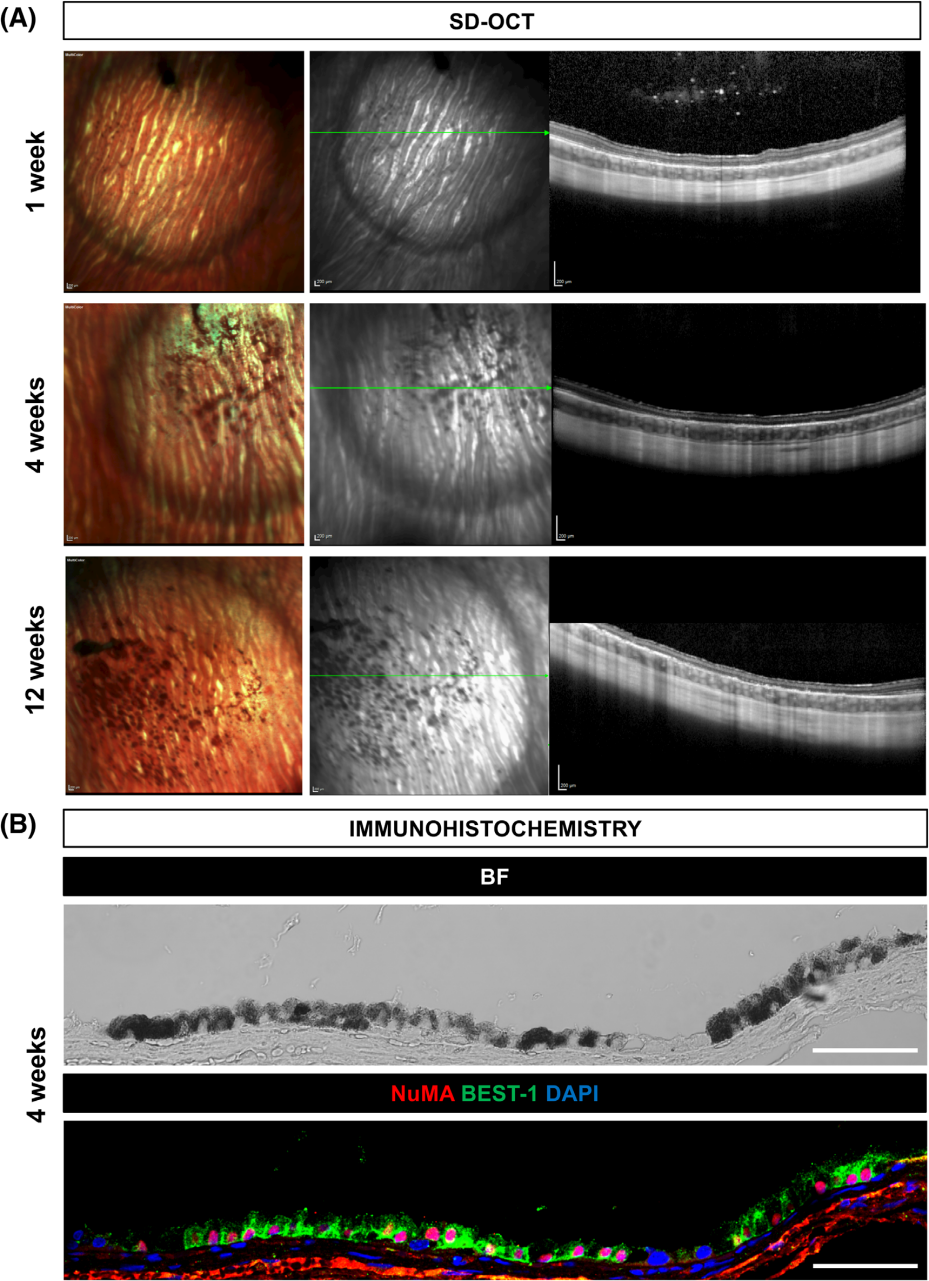
Subretinal integration of hESC‐RPE in the albino rabbit eye. A, Multicolor‐confocal scanning laser ophthalmoscopy and SD‐OCT images of representative rabbits that received hESC‐RPE cells subretinally at 1, 4, and 12 weeks after transplantation. Green lines indicate the SD‐OCT scan plane. B, Representative BF and immunofluorescent images of NuMA and BEST‐1 staining of integrated hESC‐RPE in the rabbit subretinal space at 4 weeks after transplantation. Scale bars = 200 μm (A), 50 μm (B). BF, bright‐field; hESC, human embryonic stem cell; RPE, retinal pigment epithelial

To analyze the migratory capacity of the injected cells, we first ensured that none of the genes with nonsynonymous acquired SNVs were associated with cell migration gene ontology classifications. Secondly, we evaluated the presence of human cDNA by qPCR analysis of several organs/body parts (lung, spleen, liver, heart, kidney, optic nerve, and vitreous) at different time points upon subretinal injection of 50 000 cells into albino rabbit eyes, which in turn showed absence of immune infiltration and preservation of the native retinal barrier, in addition to proper hESC‐RPE integration of pigmented human cells with expression of human NuMA and the RPE‐specific marker BEST‐1 (Figure 5; Figure S5A). Apart from evaluating transplantation at a relevant site, we also performed ectopic subcutaneous injection of 10 million cells into NOG mice to assess injection of high cell numbers. The detection limit of human cells in rabbit or mouse tissues was established to be one cell in 1.85 mg of rabbit tissue or one cell in 20 mg of mouse tissue (Figures S5B‐E). Upon injection of hESC‐RPE, all of the subcutaneously injected mouse organ samples showed undetectable levels of the human *RPLPO* transcript, and only some of the optic nerve and vitreous samples of the subretinally injected rabbits presented diverse expression levels of the same transcript (Tables 2A,B). This could be due to a sampling contamination from the transplanted hESC‐RPE located in the neighboring retina or to cells that could potentially remain in the vitreous after partial hESC‐RPE reflux observed during the subretinal injection.

**TABLE 2:** Biodistribution assessment following subretinal injections into albino rabbit eyes (A) or subcutaneous injections into NOG mice (B)

(A) Subretinal injections into albino rabbit eyes							
Threshold cycle (Ct) (hESC‐RPE/mg; hESC/mg). Detection limit: 1 cell in 1.85 mg of rabbit tissue							
	Week 1 hESC‐RPE		Week 4 hESC‐RPE		Week 12 hESC‐RPE		Month 12 hESC‐RPE
RPLPO (human)	Rabbit 1	Rabbit 2	Rabbit 3	Rabbit 4	Rabbit 5	Rabbit 6	Rabbit 7
Lung	—	—	—	—	—	—	—
Spleen	—	—	—	—	—	—	—
Liver	—	—	—	—	—	—	—
Heart	—	—	—	—	—	—	—
Kidney	—	—	—	—	—	—	—
Optic nerve	—	36.2 (2; 0.5)	—	—	—	—	—
Vitreous (eye 1)	29.9 (213; 35)	29.3 (331; 53)	29.4 (300; 48)	34.1 (8; 2)	—	32.9 (20; 4)	—
Vitreous (eye 2)	24.6 (11 282; 1245)	29.6 (262; 43)	30.7 (112; 20)	34.2 (8; 2)	34.2 (8; 2)	34.1 (8; 2)	—

(B) Subcutaneous injections into NOG mice							
Threshold cycle (C_t_) (hESC‐RPE/mg; hESC/mg). Detection limit: 1 cell in 20 mg of mouse tissue							
	Month 7 hESC‐RPE						
RPLPO (human)	Mouse 1	Mouse 2	Mouse 3	Mouse 4	Mouse 5	Mouse 6	Mouse 7
Lung	—	—	—	—	—	—	—
Spleen	—	—	—	—	—	—	—
Liver	—	—	—	—	—	—	—
Heart	—	—	—	—	—	—	—
Kidney	—	—	—	—	—	—	—
Gonads	—	—	—	—	—	—	—

Abbreviations: hESC, human embryonic stem cell; RPE, retinal pigment epithelial.

## 4 DISCUSSION

The progress in human development knowledge and the refinement in hPSC culture and differentiation protocols have brought hPSC cells and their derivatives into the first clinical trials for several diseases, including AMD.^15^, ^16^, ^17^, ^18^, ^19^, ^20^ As the use of hPSC and/or their derivatives is reaching patients, multiple efforts are put together aiming to develop tests to assess the safety of the stem cell‐derived products. Several groups have shown that hPSC may acquire genetic and/or epigenetic mutations during their culture or differentiation,^26^, ^52^ raising some concerns about their use in future cell therapies. Aiming at shedding some light on that matter, a scientific panel during ISCI 2016 (International Stem Cell Initiative) compiled risks, detection, and minimization of such mutations as well as their impact on the safety of hPSC‐derived products, resulting in preliminary guidelines followed by our study.^25^

Karyotypically variant PSCs might be associated with persistence of undifferentiated cells in xenograft tumors.^53^ Therefore, an initial test to quickly assess the genetic stability of our produced cells is to check their karyotype, which for our cells resulted in the absence of clonal aberrations. Innovations in next‐generation sequencing technologies have enabled comprehensive detection of recurrent nonrandom variations acquired during long culture conditions and present in smaller portions of the population that could provide a selective growth advantage to the cells.^26^, ^52^ We performed an extensive comparative WGS and CNV analysis between hESC‐derived RPE cells and undifferentiated hESC at different passages that allowed us to detect a relatively low number of nonrecurrent stochastic variations in our product, most probably introduced by the time that the cells have been maintained in culture rather than by the differentiation process itself. Encouraging, none of the found mutations has been located in any of the cancer‐driver genes collected in the most commonly used databases, or recently compiled list or recurrent and reportedly harmful mutations, such as p53 mutations, following extensive analysis of 140 PSC lines. Although it is important to recognize that in many cases the lack of relevant information does not allow an appropriate assessment of the potential risks. Additionally, we should also consider that these results might be subjected to some cell line to cell line variation or changes due to potential cryopreservation steps needed for a clinical GMP grade protocol. With all these in mind, our analysis could constitute the basis for future studies and a better understanding of the possible selective pressure that long‐term culture conditions could exert on stem cells‐derived products, exalting the need to develop shorter differentiation protocols. Equally important to avoid the introduction of harmful mutations during the differentiation protocol is to guarantee the quality of the starting material intended for cell therapies. The fact that our data detected SNVs reported as pathogenic in cancer‐related databases in the genome of 11 healthy people indicates that the selection of the PSC line, either embryonic or induced, should be carefully assessed before the derivation of any line for future use in the clinics.

Our findings, together with the fact that today we do not have full knowledge on the biological relevance of many mutations, accentuate the need of developing more functional assays that would be key on proving the safety of stem cell‐derived therapies. Strategies like teratoma and biodistribution studies would overcome some of the limitations observed in previously described methods, assessing any tumorigenic and/or migratory potential of the final product, and providing more relevant information of the possible impact caused by the variations.^27^, ^28^, ^29^, ^30^, ^48^ Till date, several groups leading the first clinical trials with hPSC‐RPE cells have proven the safety of their products through preclinical studies,^15^, ^16^, ^27^, ^50^, ^54^ which have been summarized in Table 1. In all these studies, they use rodent, pigs, or minipigs as preclinical models for teratoma studies with differences regarding injection site, duration, and number of animals and injected cells but overall showing that the differentiated cells fail to form any tumorigenic growths. Only da Cruz et al^15^ and Diniz et al^30^ perform more detailed studies on biodistribution to assess migration of the derived cells, also confirming its absence. The sequencing approaches used are more diverse, being Mandai et al^16^ the most exhaustive study of the iPSC‐derived cells, and concluding that no mutations in cancer‐driving genes were generally found, except for three deletions that could affect gene expression in one of the sequenced patient cells. Overall, our study is a complete work assessing both functionally and genomically the hESC‐RPE product to be delivered in a suspension format, including evaluation of teratoma formation in rodents, biodistribution/cell migration in both rodents and rabbits, and also extensive characterization of the genomic sequence comparing both the original and the differentiated material and contrasting it to both oncogenic and pathogenic databases. We show that the germline variant load is higher than the introduced variants by culture or differentiation, which suggests deeper examination of the derived products than just karyotype would be needed. In addition, our functional studies prove to be negative for teratoma and cell migration especially when integrated in the subretinal space of the rabbit eye, in line with the rest of summarized reports. Following World Health Organization's suggestions,^55^ since our product demonstrated to be stable and to not promote any tumorigenic growth or migratory behavior upon transplantation into mouse and rabbit models, the effect of the identified mutations is suggested to be innocuous. Altogether our work sets a guideline for preclinical evaluation of stem cell‐derived products both at sequence and functional levels.

It has been argued that teratoma testing with the undifferentiated PSCs would be informative to predict malignant features. With the current hESC line, we did observe yolk sac‐like structures, which also have been reported in many other PSC‐derived teratomas studies. In fact, Stevens et al observed this phenomenon in teratomas derived from normal mouse embryos when transplanted into extra‐uterine sites,^56^ also being described by the International Stem Cell Initiative in teratomas derived from multiple karyotypically normal PSC lines.^48^ Furthermore, Lim et al showed that teratocarcinoma development derived from mouse embryos injected in extra‐uterine sites is strain dependent,^57^ being NSG or NOG mice teratocarcinoma‐permissive strains. The fact that injected mature hESC‐RPE cells do not show any cancer‐related mutation and do not promote any tumor formation in NOG mice would support the idea that yolk sac formation does not always correlate with malignant properties. However, further investigations would be required to understand the significance for future clinical applications.

Finally, it should be considered that the current tumorigenic assays in animal models might not be relevant or absolutely predictive of a clinical setting as the immunological response could be very different in a xenogeneic situation and in immune deficient rodent animals. Our data further show that fairly large number of undifferentiated cells (up to 10 000 hESCs) even fail to induce a tumorigenic growth. For this purpose, detailed characterization of lingering undifferentiated or even partially differentiated cells using single‐cell analysis techniques such as flow cytometry and transcriptomics may give a much more sensitive and informative readout in that regard. Furthermore, a tumorigenic mutation may not manifest during relatively short‐term tumorigenic assays but could instead be picked up through WGS. The relevance of the costly and time consuming in vivo tumorigenic studies should be discussed and evaluated with regulatory bodies and at least be contrasted with other methodologies to characterize the safety of any stem cell‐derived cellular product.

## 5 CONCLUSION

In the present study, we show that our differentiation protocol^7^ generates pure, safe, and stable hESC‐RPE cells without any abnormal chromosomal organization nor carcinogenic mutation load at a SNV or CNV level. In addition, we demonstrate that our hESC‐RPE cells do not form any tumorigenic structures after 7 months when injected subcutaneously in immunodeficient mice, neither migrate to other organs in mouse or rabbit models. Even though functional assays like tumorigenicity and biodistribution studies are considered gold standard studies to prove the safety of these therapies, we would like to argue that comparative genome‐wide genomic analysis together with single‐cell characterization using flow cytometry and transcriptional analysis may be equally or even more informative when developing and testing new hPSC‐derived therapies.

## CONFLICT OF INTEREST

The authors declared no potential conflicts of interest.

## AUTHOR CONTRIBUTIONS

S.P.‐R., P.K., S.P.S.: conception and design, collection and assembly of data, data analysis and interpretation, manuscript writing, final approval of manuscript; M.A.: contributed to animal experiments; H.A.: conception and design, data analysis and interpretation; H.B.: contributed to animal experiments, conception and design, data analysis and interpretation; A.P.R.: collection and assembly of data; E.F.N.: sharing of reagents; A.K., F.L.: conception and design, data analysis and interpretation, manuscript writing and final approval of manuscript.

## Supporting information


**Appendix** **S1**: Supporting Information

## Data Availability

The data that support the findings of this study are available on request from the corresponding author.
