# Supplementary material for: Preclinical safety studies of human embryonic stem cell‐derived retinal pigment epithelial cells for the treatment of age‐related macular degeneration
Source: Stem Cells Transl Med. 2020 Apr 22;9(8):936–53. doi: 10.1002/sctm.19-0396 (PMC7381808; doi:10.1002/sctm.19-0396)
Supplement: Supplementary file 1 — Appendix S1: Supporting Information [file SCT3-9-936-s001.pdf]

SUPPLEMENTAL INFORMATION

Preclinical safety studies of human embryonic stem cell-derived retinal pigment epithelial cells for the treatment of age-related macular degeneration

Petrus-Reurer et al.

Supplemental Figures S1 – S5

Supplemental Tables S1 – S7

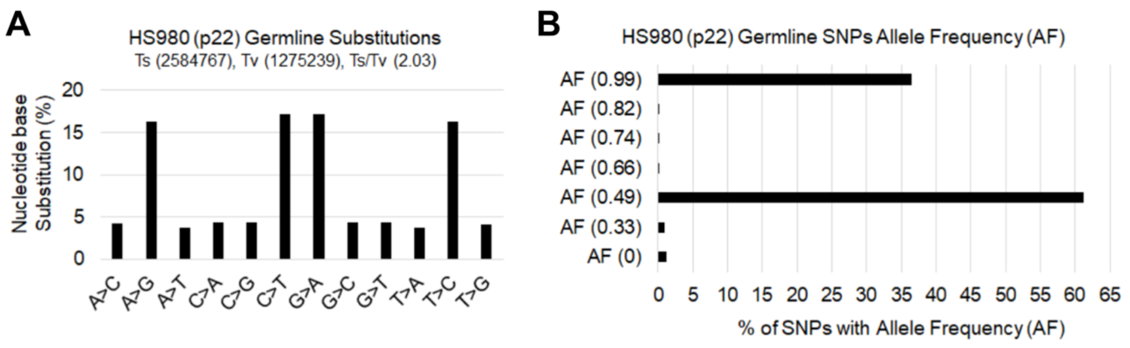

SUPPLEMENTAL FIGURE S1. Annotation of germline SNVs.

(A) Bar charts showing the relative percentage of mutational subtypes for the germline SNVs in HS980 (p22) sample. **(B)** Bar charts showing the relative percentage of germline SNP allele frequency for the HS980 (p22) sample.

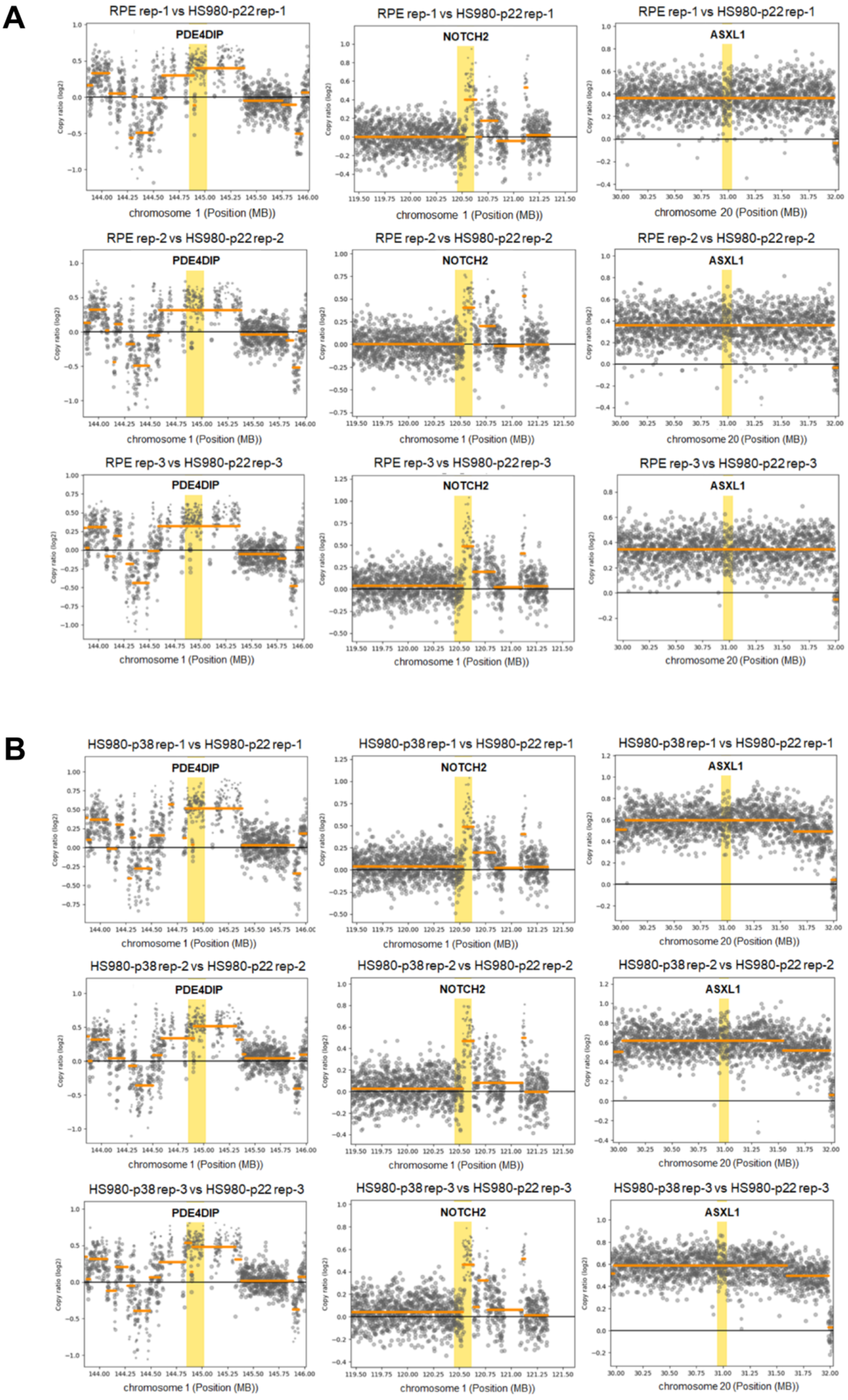
A
RPE rep-1 vs HS980-p22 rep-1
PDE4DIP
NOTCH2
ASXL1
RPE rep-2 vs HS980-p22 rep-2
RPE rep-3 vs HS980-p22 rep-3
Copy ratio (log2)
chromosome 1 (Position (MB))
chromosome 20 (Position (MB))
B
HS980-p38 rep-1 vs HS980-p22 rep-1
HS980-p38 rep-2 vs HS980-p22 rep-2
HS980-p38 rep-3 vs HS980-p22 rep-3
PDE4DIP
NOTCH2
ASXL1

SUPPLEMENTAL FIGURE S2. Copy number profile for cancer-driver genes (*PDE4DIP, NOTCH2 and ASXL1*) in hESC-RPE and HS980 (p38) samples.

(A) Plots showing copy number ratio by CNVkit for cancer-driver genes *PDE4DIP, NOTCH2* and *ASXL1* for hESC-RPE samples. X-axis represents log2 copy ratio of hESC-RPE samples compared with their respective HS980 (p22) control samples and Y-axis represents genomic position. **(B)** Plots showing copy number ratio by CNVkit for *PDE4DIP, NOTCH2* and *ASXL1* genes for HS980 (p38) samples. X-axis represents log2 copy ratio of HS980 (p38) samples compared with their respective HS980 (p22) control samples and Y-axis represents genomic position.

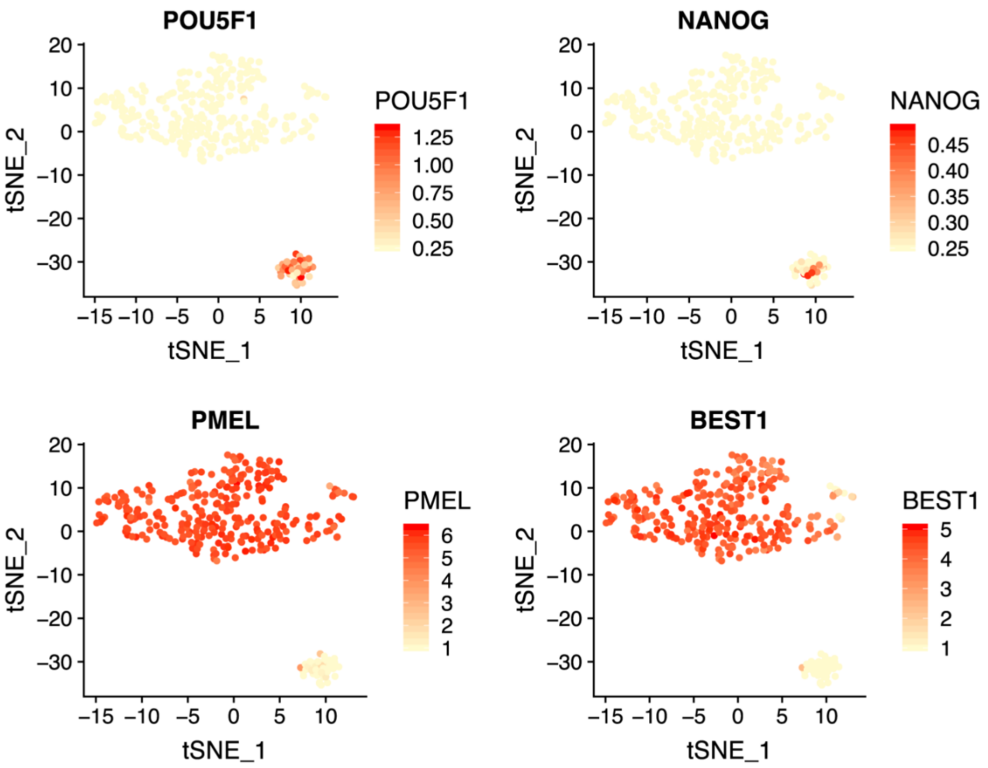

SUPPLEMENTAL FIGURE S3. Single cell RNA sequencing analysis of hESC-RPE and hESC.

tSNE plots showing expression of selective hESC and RPE markers in hESC and hESC-RPE cell clusters.

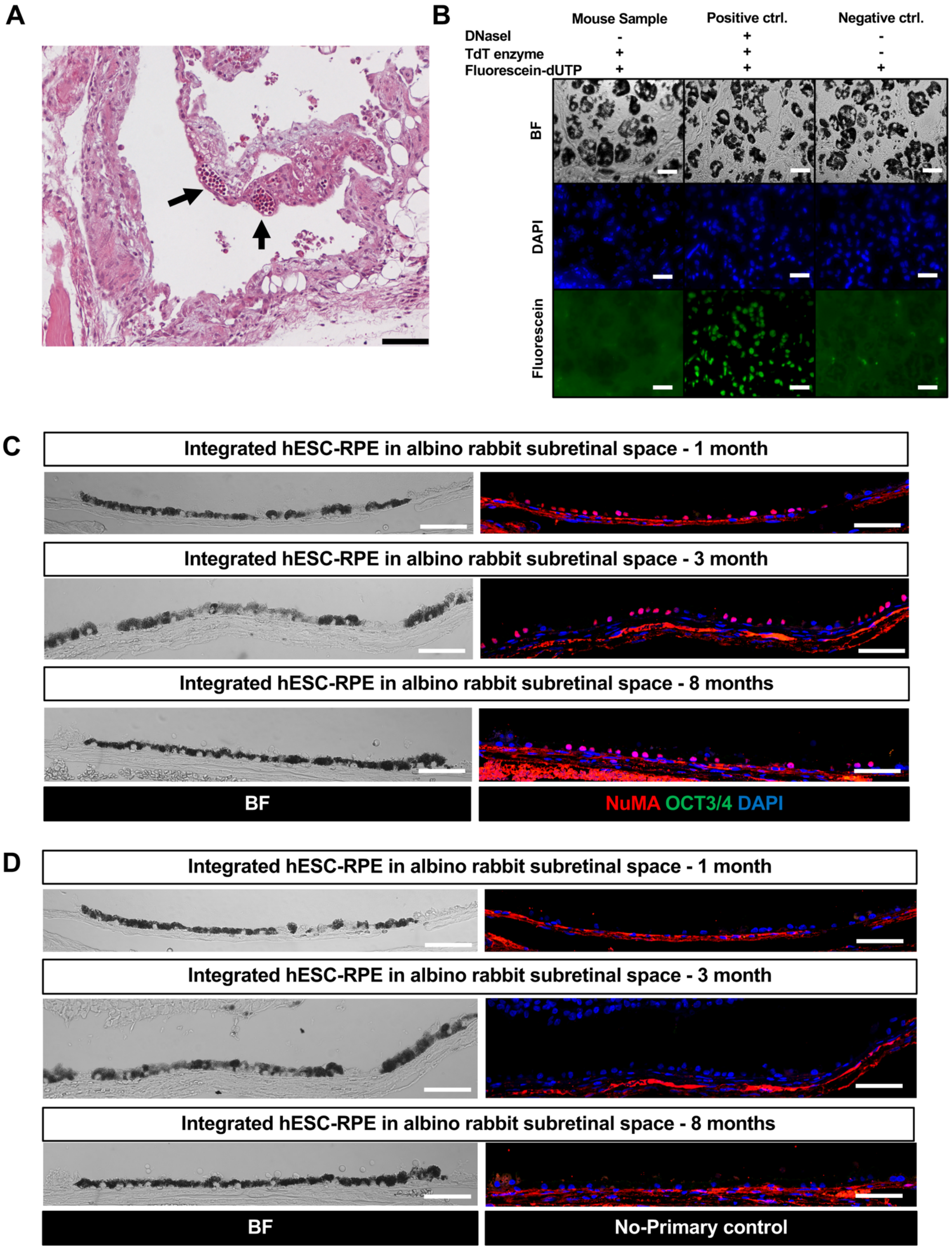
A
B
Mouse Sample
Positive ctrl.
Negative ctrl.
DNaseI
TdT enzyme
Fluorescein-dUTP
BF
DAPI
Fluorescein
C
Integrated hESC-RPE in albino rabbit subretinal space - 1 month
Integrated hESC-RPE in albino rabbit subretinal space - 3 month
Integrated hESC-RPE in albino rabbit subretinal space - 8 months
BF
NuMA OCT3/4 DAPI
D
Integrated hESC-RPE in albino rabbit subretinal space - 1 month
Integrated hESC-RPE in albino rabbit subretinal space - 3 month
Integrated hESC-RPE in albino rabbit subretinal space - 8 months
BF
No-Primary control

SUPPLEMENTAL FIGURE S4. Evaluation of hESC-RPE injected subcutaneously into NOG mice or subretinally into albino rabbits.

(A) HE staining image showing dilated vascular profiles densely packed with nucleated cells with an intensely eosinophilic cytoplasm representing primitive hematopoiesis. Cells with hepatoid features border these yolk sac blood islands. **(B)** Representative BF and immunofluorescence images showing TUNEL-negative (alive) collected hESC-RPE cells 7 months after subcutaneous transplantation into NOG mice. **(C)** Representative BF and immunofluorescent images of NuMA and OCT3/4 staining of integrated hESC-RPE in the rabbit subretinal space at 1, 3 and 8 months after transplantation. **(D)** No-primary control stainings of the same specimens showed in Figure S4C.

Scale bars: (A, B) = 100 μm; (C, D) = 50 μm

A

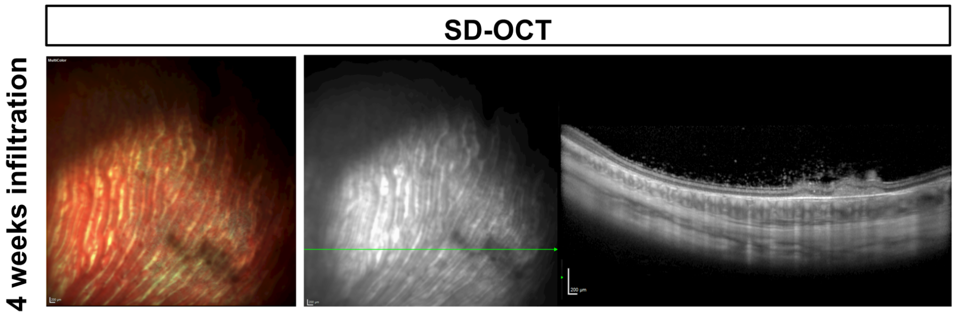

B

Nr hESC	Cells/mg	Log (Cells/mg)	Ct RPLPO (Human)
Rb Ki_10	0.54	-0.27	36.15
Rb Ki_100	5.4	0.73	32.11
Rb Ki_1000	54	1.73	29.38
Rb Ki_10000	540	2.73	26.70
Rb Ki_100000	5400	3.73	21.61
Rb Ki_1000000	54000	4.73	19.28

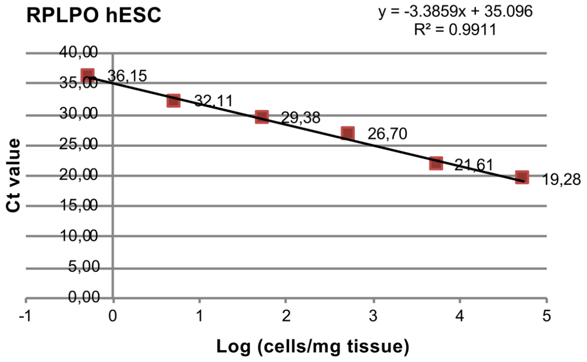

C

Nr hESC-RPE	Cells/mg	Log (Cells/mg)	Ct RPLPO (Human)
Rb Ki_10	0.54	-0.27	36.26
Rb Ki_100	5.4	0.73	35.97
Rb Ki_1000	54	1.73	32.14
Rb Ki_10000	540	2.73	29.40
Rb Ki_100000	5400	3.73	24.90
Rb Ki_1000000	54000	4.73	22.19

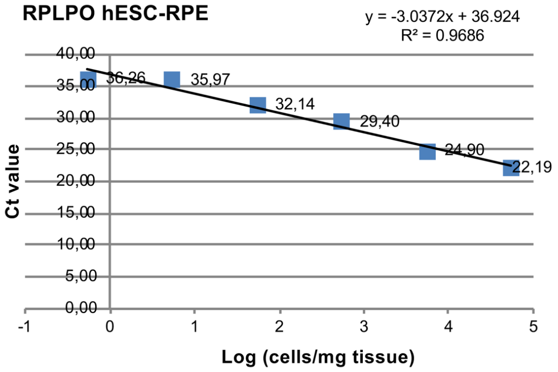

Detection limit: 1 cell in 1.85mg of rabbit tissue

D

Nr hESC	Cells/mg	Log (Cells/mg)	Ct RPLPO (Human)
Ms WT Li_10	0.5	-0.30	33.03
Ms WT Li_100	5	0.69	29.16
Ms WT Li_1000	50	1.69	25.31
Ms WT Li_10000	500	2.69	22.15
Ms WT Li_100000	5000	3.69	18.66

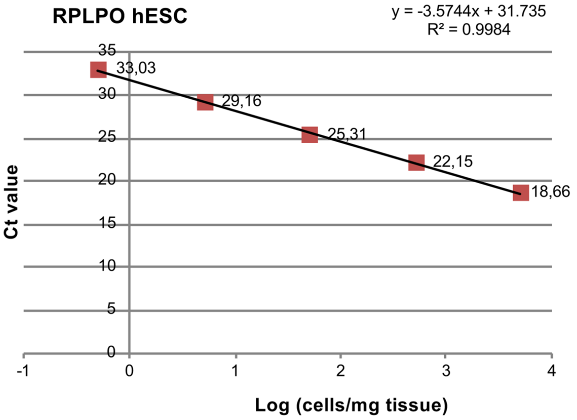

E

Nr hESC-RPE	Cells/mg	Log (Cells/mg)	Ct RPLPO (Human)
Ms WT Li_1	0.05	-1.30	34.49
Ms WT Li_10	0.5	-0.30	32.13
Ms WT Li_100	5	0.69	28
Ms WT Li_1000	50	1.69	27.72
Ms WT Li_10000	500	2.69	24.55
Ms WT Li_100000	5000	3.69	21.13
Ms WT Li_1000000	50000	4.69	20.36

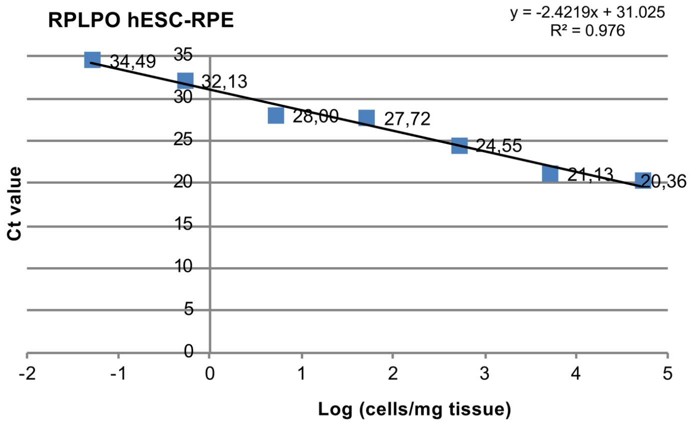

Detection limit: 1 cell in 20mg of mouse tissue

SUPPLEMENTAL FIGURE S5. hESC and hESC-RPE cell spiking in rabbit and mouse tissues for biodistribution studies.

(A) SD-OCT image showing infiltration in the subretinal space 4 weeks after injection of hESC-RPE cells. **(B)** Detection of RPLPO transcripts by qPCR after serial dilutions of hESC in rabbit (Rb) kidney (Ki) tissue. **(C)** Detection of RPLPO transcripts by qPCR after serial dilutions of hESC-RPE in rabbit kidney tissue. **(D)** Detection of RPLPO transcripts by qPCR after serial dilutions of hESC in wild-type (WT) mouse (Ms) liver (Li) tissue. **(E)** Detection of RPLPO transcripts by qPCR after serial dilutions of hESC-RPE in wild-type mouse liver tissue.

Nr: Number

Scale bars: (A) = 200 µm

Supplemental Table S1. Tumorigenicity studies design. Relation of injected cell type, number of cells and monitoring time in each group of NOG mice.

Mice group	Number of mice	Cell type	Number of cells	Monitoring period
1	10	hESC	$1x10^{1}$	4 months
2	10	hESC	$1x10^{2}$	4 months
3	10	hESC	$1x10^{3}$	4 months
4	10	hESC	$1x10^{4}$	4 months
5	10	hESC	$1x10^{5}$	4 months
6	10	hESC	$1x10^{6}$	4 months
7	10	3-week EBs	$1x10^{7}$	7 months
8	10	5-week EBs	$1x10^{7}$	7 months
9	10	hESC-RPE	$1x10^{7}$	7 months

Supplemental Table S2. Whole-genome DNA sequence alignment statistics for HS980 (p22), hESC-RPE and HS980 (p38) samples.

Sample	Total Reads (paired-end)	Aligned Reads (both in pairs)	Coverage (mean)	Non-duplicated (read pairs)
HS980 p22 rep-1	824,883,240	812,036,402(98.44%)	38.24	352,832,529(84.7%)
HS980 p22 rep-1	839,393,930	832,313,573(99.16%)	39.27	360,164,708(85.3%)
HS980 p22 rep-1	787,107,006	782,885,654(99.46%)	36.91	362,620,161(91.9%)
hESC-RPE rep-1	834,164,276	793,489,002(95.12%)	37.56	364,737,787(83.6%)
hESC-RPE rep-2	768,238,398	764,986,043(99.58%)	36.26	354,434,223(92.1%)
hESC-RPE rep-3	836,031,077	830,641,424(99.36%)	39.12	376,953,431(89.1%)
HS980 p38 rep-1	831,868,835	824,536,555(99.12%)	38.96	335,881,565(80.3%)
HS980 p38 rep-2	788,437,294	783,554,320(99.38%)	37.06	362,663,677(91.7%)
HS980 p38 rep-3	775,465,729	771,450,994(99.48%)	36.63	359,163,976(92.4%)

Supplemental Table S3. HS980 (p22) Germline SNV overlapping to ClinVar Database.

# HS980 p22 combined all three replicates germline SNVs
SNVs overlapping to ClinVar clinical Databases (release 29th October 2017)
# SNVs reported as CLNSIG (clinical significance) ="Pathogenic"
CLNDN; Description="ClinVar's preferred disease name for the concept specified by disease identifiers in CLNDISDB"
GMAF = Global Minor Allele Frequency
Common Variant (minor allele frequency (GMAF) of >= 0.01 in at least one 1000 Genomes Phase III major population with at least two individuals from different families having same minor allele)

Chr_No	Position	dbSNP_rs-ID	ClinVar-ID	REF	ALT	GMAF	Population Status	Gene-ID	CLNSIG	CLNDN
chr1	55504650	rs2479409	440703	G	A	0.3986	Common Variant	PCSK9	Benign/Pathogenic	Familial_hypercholesterolemia
chr1	198796120	rs12406470	427753	C	T	0.4816	Common Variant	MIR181A1HG	Pathogenic	Acute_myeloid_leukemia_with_maturation
chr1	198867678	rs60639710	427754	G	T	0.2043	Common Variant	MIR181A1HG	Pathogenic	Acute_myeloid_leukemia_with_maturation
chr1	198868084	rs10800597	427755	G	A	0.2045	Common Variant	MIR181A1HG	Pathogenic	Acute_myeloid_leukemia_with_maturation
chr1	198869514	rs10800598	427756	T	C	0.1799	Common Variant	MIR181A1HG	Pathogenic	Acute_myeloid_leukemia_with_maturation
chr4	6295693	rs6446482	4528	C	G	0.2788	Common Variant	WFS1	Pathogenic	Diabetes_mellitus,_noninsulin-dependent
chr4	187158034	rs3733402	12037	G	A	0.3954	Common Variant	KLKB1	Pathogenic	Prekallikrein_deficiency
chr5	176520243	rs351855	16326	G	A	0.2995	Common Variant	FGFR4	Pathogenic	Cancer_progression_and_tumor_cell_motility
chr8	11606312	rs3735819	433016	T	C	0.1284	Common Variant	GATA4	Pathogenic	Congenital_heart_disease
chr8	11606364	rs10503425	433017	G	C	0.0493	Common Variant	GATA4	Pathogenic	Congenital_heart_disease
chr8	11614769	rs12156163	433023	C	T	0.1142	Common Variant	GATA4	Pathogenic	Congenital_heart_disease
chr8	11615695	rs745379	433015	A	G	0.2702	Common Variant	GATA4	Pathogenic	Congenital_heart_disease
chr8	11616836	rs804290	433024	G	A	0.117	Common Variant	GATA4	Pathogenic	Congenital_heart_disease
chr8	11617240	rs12458	433026	A	T	0.4	Common Variant	GATA4	Pathogenic	Congenital_heart_disease
chr10	54531235	rs1800450	14350	C	T	0.122	Common Variant	MBL2	Pathogenic	Mannose-binding_protein_deficiency
chr11	48145375	rs1566734	8690	A	C	0.1899	Common Variant	PTPRJ	Pathogenic	Carcinoma_of_colon
chr16	16291858	rs12929920	433383	G	C	0.2686	Common Variant	ABCC6	Benign/Pathogenic	Pseudoxanthoma_elasticum
chr16	16291871	rs9940089	433382	G	C	0.2121	Common Variant	ABCC6	Benign/Pathogenic	Pseudoxanthoma_elasticum
chr17	32579788	rs1024611	14207	A	G	0.3636	Common Variant	CCL2	Pathogenic	Coronary_artery_disease
chr20	23618427	rs1064039	5635	C	T	0.2123	Common Variant	CST3	Pathogenic	Age-related_macular_degeneration_11

Highlighted in grey: genes with some clinical relevance reported for AMD in ClinVar

Supplemental Table S4A. HS980 (p22) Germline SNV overlapping to COSMIC Cancer Gene Census.

HS980 p22 Germline SNVs common to three replicates
SNVs reported in COSMIC database (Catalog of Somatic Mutation in Cancer - release v83; Coding; 07-11-2017)
SNVs with "FATHMM" prediction as "Pathogenic"
# SNVs within Cancer Driver Genes (COSMIC Cancer Gene Census)
GMAF in population = Global Minor Allele Frequency
Common Variant (minor allele frequency (GMAF) of >= 0.01 in at least one 1000 Genomes Phase III major population)

Chr_No	Position	dbSNP_rs-ID	COSMIC-ID	REF	ALT	GMAF	Population Status	FATHMM SCORE	Gene-ID	BASE CHANGE	AA CHANGE	Substitution Type
chr2	29416481	rs1881420	COSM1130802	T	C	0.4151	Common Variant	0.92214	ALK	c.4472A>G	p.K1491R	Missense
chr2	29940529	rs2246745	COSM4416269	A	T	0.4107	Common Variant	0.9054	ALK	c.702T>A	p.P234P	coding silent
chr2	141116447	rs35546150	COSM5019952	G	T	0.0449	Common Variant	0.86876	LRP1B	c.11200C>A	p.Q3734K	Missense
chr2	141274576	rs4954672	COSM4001201	T	C	0.6424	Common Variant	0.93919	LRP1B	c.8031A>G	p.Q2677Q	coding silent
chr2	212587119	rs77309171	COSM4583147	T	C	0.002	Common Variant	0.86171	ERBB4	c.882A>G	p.P294P	coding silent
chr3	37053568	rs1799977	COSM1131469	A	G	0.1296	Common Variant	0.80196	MLH1	c.655A>G	p.I219V	Missense
chr3	128204951	rs2335052	COSM445531	C	T	0.2328	Common Variant	0.91046	GATA2	c.490G>A	p.A164T	Missense
chr4	55152040	rs2228230	COSM22413	C	T	0.2404	Common Variant	0.88315	PDGFRA	c.2472C>T	p.V824V	coding silent
chr4	55979558	rs2305948	COSM1131107	C	T	0.1526	Common Variant	0.96877	KDR	c.889G>A	p.V297I	Missense
chr4	187630590	rs3733415	COSM4416272	G	A	0.2614	Common Variant	0.95514	FAT1	c.392C>T	p.A131V	Missense
chr5	56177443	rs702689	COSM4003565	G	A	0.4764	Common Variant	0.98239	MAP3K1	c.1927G>A	p.D643N	Missense
chr5	56177743	rs832582	COSM4416191	G	A	0.7083	Common Variant	0.76719	MAP3K1	c.2227G>A	p.V743I	Missense
chr5	112162854	rs2229992	COSM1432175	T	C	0.51	Common Variant	0.83456	APC	c.1458T>C	p.Y486Y	coding silent
chr5	112176559	rs866006	COSM6475354	T	G	0.333	Common Variant	0.71742	APC	c.5268T>G	p.S1756S	coding silent
chr7	55249063	rs1050171	COSM1451600	G	A	0.4327	Common Variant	0.95009	EGFR	c.2361G>A	p.Q787Q	coding silent
chr7	116340262	rs33917957	COSM5020653	A	G	0.0329	Common Variant	0.86365	MET	c.1124A>G	p.N375S	Missense
chr7	128845511	rs111694017	COSM1738148	G	A	0.0024	Common Variant	0.90625	SMO	c.808G>A	p.V270I	Missense
chr7	138556013	rs776449063	COSM5434577	G	A	0.00022(ExAC)	NOT Common	0.84266	KIAA1549	c.4441C>T	p.R1481W	Missense
chr8	69020496	rs4260880	COSM3763384	T	C	0.4798	Common Variant	0.85007	PREX2	c.2868T>C	p.S956S	coding silent
chr9	117846570	rs2274836	COSM3763575	C	T	0.4641	Common Variant	0.72876	TNC	c.2049G>A	p.E683E	coding silent
chr9	135985796	rs3761824	COSM4163477	C	T	0.2486	Common Variant	0.97073	RALGDS	c.372G>A	p.V124V	coding silent
chr11	102201848	rs17878663	COSM3998183	G	A	0.0699	Common Variant	0.70352	BIRC3	c.1200G>A	p.Q400Q	coding silent
chr12	25362777	rs1137282	COSM3753105	A	G	0.1755	Common Variant	0.76767	KRAS	c.519T>C	p.D173D	coding silent
chr12	121416650	rs1169288	COSM430522	A	C	0.2985	Common Variant	0.91018	HNF1A	c.79A>C	p.I27L	Missense
chr12	121435342	rs2259820	COSM3931546	C	T	0.3167	Common Variant	0.9731	HNF1A	c.1375C>T	p.L459L	coding silent
chr12	121435427	rs2464196	COSM4984989	G	A	0.3177	Common Variant	0.82717	HNF1A	c.1460G>A	p.S487N	Missense
chr13	21562948	rs558614	COSM432208	G	A	0.3704	Common Variant	0.96847	LATS2	c.971C>T	p.A324V	Missense
chr13	28624294	rs1933437	COSM5019176	G	A	0.5587	Common Variant	0.92594	FLT3	c.680C>T	p.T227M	Missense
chr14	38061742	rs7144658	COSM3753953	C	T	0.417	Common Variant	0.83972	FOXA1	c.247G>A	p.A83T	Missense
chr14	105239894	rs1130233	COSM3765730	C	T	0.3225	Common Variant	0.71625	AKT1	c.726G>A	p.E242E	coding silent
chr16	9943666	rs2229193	COSM3999994	C	T	0.2258	Common Variant	0.73792	GRIN2A	c.1275G>A	p.L425L	coding silent
chr19	42799049	rs1052023	COSM3756833	C	T	0.1198	Common Variant	0.78474	CIC	c.4533C>T	p.I1511I	coding silent
chr20	40714479	rs2016647	COSM3758574	G	A	0.144	Common Variant	0.91263	PTPRT	c.3927C>T	p.Y1309Y	coding silent
chrX	44938563	rs20539	COSM1179848	G	A	0.2217	Common Variant	0.88609	KDM6A	c.3111G>A	p.Q1037Q	coding silent
chrX	153629155	rs4909	COSM4590446	A	G	0.6689	Common Variant	0.81036	RPL10	c.605A>G	p.N202S	Missense

Highlighted in grey: genes with SNV non-common to population

Supplemental Table S4B. HS980 (p22) Germline SNV overlapping to Bailey MH et al, Cancer Driver Genes.

HS980 p22 Germline SNVs common to three replicates
SNVs reported in COSMIC database (Catalog of Somatic Mutation in Cancer - release v83; Coding; 07-11-2017)
SNVs with "FATHMM" prediction as "Pathogenic"
# SNVs within Cancer Driver Genes (Bailey MH et al.; Cell-2018; PMID: 29625053).
GMAF = Global Minor Allele Frequency
Common Variant (minor allele frequency (GMAF) of >= 0.01 in at least one 1000 Genomes Phase III major population)

Chr_No	Position	dbSNP_rs-ID	COSMIC-ID	REF	ALT	GMAF	Population Status	FATHMM Score	Gene-ID	BASE Change	AA Change	Substitution Type
chr2	29416481	rs1881420	COSM1130802	T	C	0.4151	Common Variant	0.92214	ALK	c.4472A>G	p.K1491R	Missense
chr2	29940529	rs2246745	COSM4416269	A	T	0.411	Common Variant	0.9054	ALK	c.702T>A	p.P234P	coding silent
chr2	212587119	rs77309171	COSM4583147	T	C	0.002	Common Variant	0.86171	ERBB4	c.882A>G	p.P294P	coding silent
chr3	37053568	rs1799977	COSM1131469	A	G	0.1296	Common Variant	0.80196	MLH1	c.655A>G	p.I219V	Missense
chr4	55152040	rs2228230	COSM22413	C	T	0.2404	Common Variant	0.88315	PDGFRA	c.2472C>T	p.V824V	coding silent
chr4	187630590	rs3733415	COSM4416272	G	A	0.2614	Common Variant	0.95514	FAT1	c.392C>T	p.A131V	Missense
chr5	112162854	rs2229992	COSM1432175	T	C	0.51	Common Variant	0.83456	APC	c.1458T>C	p.Y486Y	coding silent
chr5	56177443	rs702689	COSM4003565	G	A	0.4764	Common Variant	0.98239	MAP3K1	c.1927G>A	p.D643N	Missense
chr5	56177743	rs832582	COSM4416191	G	A	0.7083	Common Variant	0.76719	MAP3K1	c.2227G>A	p.V743I	Missense
chr5	112176559	rs866006	COSM6475354	T	G	0.333	Common Variant	0.71742	APC	c.5268T>G	p.S1756S	coding silent
chr7	55249063	rs1050171	COSM1451600	G	A	0.4327	Common Variant	0.95009	EGFR	c.2361G>A	p.Q787Q	coding silent
chr7	116340262	rs33917957	COSM5020653	A	G	0.0329	Common Variant	0.86365	MET	c.1124A>G	p.N375S	Missense
chr12	25362777	rs1137282	COSM3753105	A	G	0.1755	Common Variant	0.76767	KRAS	c.519T>C	p.D173D	coding silent
chr13	21562948	rs558614	COSM432208	G	A	0.37	Common Variant	0.96847	LATS2	c.971C>T	p.A324V	Missense
chr13	28624294	rs1933437	COSM5019176	G	A	0.5587	Common Variant	0.92594	FLT3	c.680C>T	p.T227M	Missense
chr14	38061742	rs7144658	COSM3753953	C	T	0.417	Common Variant	0.83972	FOXA1	c.247G>A	p.A83T	Missense
chr14	105239894	rs1130233	COSM3765730	C	T	0.3225	Common Variant	0.71625	AKT1	c.726G>A	p.E242E	coding silent
chr19	42799049	rs1052023	COSM3756833	C	T	0.1198	Common Variant	0.78474	CIC	c.4533C>T	p.I1511I	coding silent
chrX	44938563	rs20539	COSM1179848	G	A	0.2217	Common Variant	0.88609	KDM6A	c.3111G>A	p.Q1037Q	coding silent
chrX	32380996	rs1801187	COSM4999535	C	T	0.4652	Common Variant	0.96068	DMD	c.1211G>A	p.R404H	Missense

Supplemental Table S4C. HS980 (p22) Germline SNV overlapping to Shibata Cancer Driver Genes.

HS980 p22 Germline SNVs common to three replicates.
SNVs reported in COSMIC database (Catalog of Somatic Mutation in Cancer - release v83; Coding; 07-11-2017)
# SNVs with "FATHMM" prediction as "Pathogenic"
# SNVs within Cancer Driver Genes (Shibata Cancer driver gene list based on an article (Cancer Research 72:636-644, 2012)).
GMAF = Global Minor Allele Frequency
Common Variant (minor allele frequency (GMAF) of >= 0.01 in at least one 1000 Genomes Phase III major population)

Chr_No	Position	dbSNP_rs-ID	COSMIC-ID	REF	ALT	GMAF	Population Status	FATHMM SCORE	Gene-ID	BASE CHANGE	AA CHANGE	Substitution Type
chr2	29416481	rs1881420	COSM1130802	T	C	0.4151	Common Variant	0.92214	ALK	c.4472A>G	p.K1491R	Missense
chr2	29940529	rs2246745	COSM4416269	A	T	0.411	Common Variant	0.9054	ALK	c.702T>A	p.P234P	Coding silent
chr3	37053568	rs1799977	COSM1131469	A	G	0.1296	Common Variant	0.80196	MLH1	c.655A>G	p.I219V	Missense
chr4	55979558	rs2305948	COSM1131107	C	T	0.1526	Common Variant	0.96877	KDR	c.889G>A	p.V297I	Missense
chr4	55152040	rs2228230	COSM22413	C	T	0.2404	Common Variant	0.88315	PDGFRA	c.2472C>T	p.V824V	Coding silent
chr5	112162854	rs2229992	COSM1432175	T	C	0.51	Common Variant	0.83456	APC	c.1458T>C	p.Y486Y	Coding silent
chr5	112176559	rs866006	COSM6475354	T	G	0.333	Common Variant	0.71742	APC	c.5268T>G	p.S1756S	Coding silent
chr7	55249063	rs1050171	COSM1451600	G	A	0.4327	Common Variant	0.95009	EGFR	c.2361G>A	p.Q787Q	Coding silent
chr7	128845511	rs111694017	COSM1738148	G	A	0.0024	Common Variant	0.90625	SMO	c.808G>A	p.V270I	Missense
chr7	116340262	rs33917957	COSM5020653	A	G	0.0329	Common Variant	0.86365	MET	c.1124A>G	p.N375S	Missense
chr12	25362777	rs1137282	COSM3753105	A	G	0.1755	Common Variant	0.76767	KRAS	c.519T>C	p.D173D	Coding silent
chr12	121435342	rs2259820	COSM3931546	C	T	0.3167	Common Variant	0.9731	HNF1A	c.1375C>T	p.L459L	Coding silent
chr12	121416650	rs1169288	COSM430522	A	C	0.2985	Common Variant	0.91018	HNF1A	c.79A>C	p.I27L	Missense
chr12	121435427	rs2464196	COSM4984989	G	A	0.3177	Common Variant	0.82717	HNF1A	c.1460G>A	p.S487N	Missense
chr13	28624294	rs1933437	COSM5019176	G	A	0.5587	Common Variant	0.92594	FLT3	c.680C>T	p.T227M	Missense
chr14	105239894	rs1130233	COSM3765730	C	T	0.3225	Common Variant	0.71625	AKT1	c.726G>A	p.E242E	Coding silent
chr19	42799049	rs1052023	COSM3756833	C	T	0.1198	Common Variant	0.78474	CIC	c.4533C>T	p.I1511I	Coding silent
chrX	44938563	rs20539	COSM1179848	G	A	0.2217	Common Variant	0.88609	KDM6A	c.3111G>A	p.Q1037Q	Coding silent

Supplemental Table S5. Exonic and Spliced somatic SNVs for hESC-RPE samples compared with respective HS980 (p22) samples.

Somatic SNVs Identified using GATK-MuTect2 (HS980 p22 vs RPE)
Combined Non-redundant SNVs (replicate-1, 2 & 3)
ANNOVAR annotation of SNVs
SNVs with Missence, Frameshift and Silent mutation annotation
Average Expression (RPKM) of gene from single-cell experiment (hES cells & RPE cells)

Chr_No	Start	End	Ref	Alt	dbSNP_rs-ID	Gene-ID	Annotation	Amino Acid Change	Avg. Exp. hESC	Avg. Exp. RPE
chr1	17086940	17086941	AC	-	rs748266310	MST1L	Frameshift Deletion	c.383_384del:p.128_128del	0.80	0.03
chr11	56143716	56143719	TGTT	-	rs760647141	OR8U1	Frameshift Deletion	c.617_620del:p.206_207del	0.00	0.00
chr1	145349584	145349584	T	A	rs370191373	NBPF10	Non-synonymous	c.T6999A:p.D2333E,	0.03	1.14
chr1	152186042	152186042	A	G	rs12751022	HRNR	Non-synonymous	c.T8063C:p.L2688S	0.05	0.01
chr12	10588530	10588530	C	G	rs34195537	KLRC2	Non-synonymous	c.G56C:p.R19P	0.00	0.00
chr16	21623970	21623970	A	T	.	METTL9	Non-synonymous	c.A170T:p.Y57F	73.69	114.00
chr19	54745682	54745682	C	T	rs111666280	LILRA6	Non-synonymous	c.G419A:p.R140Q	0.00	0.00
chr2	113147370	113147370	G	A	rs202082997	RGPD5	Non-synonymous	c.C3152T:p.T1051I	0.00	0.00
chr22	24579049	24579049	G	A	rs35660748	SUSD2	Non-synonymous	c.G101A:p.R34H	0.24	0.80
chr3	47030858	47030858	C	T	rs764816462	NBEAL2	Non-synonymous	c.C460T:p.R154C	0.00	0.14
chr6	30954694	30954694	C	A	rs112415706	MUC21	Non-synonymous	c.C742A:p.P248T	0.00	0.00
chr7	100639618	100639618	C	T	rs111933539	MUC12	Non-synonymous	c.C5774T:p.T1925M	0.14	0.07
chrX	57618621	57618621	T	C	rs113289397	ZXDB	Non-synonymous	c.T140C:p.L47P	1.46	2.16
chr1	201180222	201180222	G	A	rs28465285	IGFN1	Synonymous	c.G6201A:p.E2067E	0.00	0.02
chr11	1092684	1092684	C	T	rs201269049	MUC2	Synonymous	c.C4503T:p.S1501S	0.00	3.88
chr11	117077034	117077034	A	G	rs28590104	PCSK7	Synonymous	c.T2037C:p.T679T	6.60	4.13
chr17	15539560	15539560	C	T	rs9911397	TRIM16	Synonymous	c.G639A:p.A213A	0.89	4.66
chr17	39197593	39197593	G	T	rs145621540	KRTAP1-1	Synonymous	c.C57A:p.T19T	0.00	0.00
chr18	14542867	14542867	C	T	rs200779556	POTEC	Synonymous	c.G279A:p.T93T	0.00	0.00
chr20	60904081	60904081	G	A	rs544096167	LAMA5	Synonymous	c.C4266T:p.N1422N	4.91	11.93
chr4	367291	367291	T	C	.	ZNF141	Synonymous	c.T1065C:p.N355N	17.45	17.16
chr9	43628651	43628651	A	C	rs2261119	SPATA31A6	Synonymous	c.T291G:p.L97L	0.00	0.00
chrX	140785790	140785790	T	C	rs139363903	SPANXD	Synonymous	c.A126G:p.L42L	0.00	0.00
chr1	16890602	16890602	T	G	rs2990551	NBPF1	Unknown	UNKNOWN	0.34	2.32
chr1	148017582	148017582	A	C	rs879951427	NBPF8	Unknown	UNKNOWN	0.05	2.86

Highlighted in grey: genes with some clinical relevance reported in COSMIC/ClinVar

Supplemental Table S6. Exonic and Spliced somatic SNVs for HS980 (p38) samples compared with respective HS980 (p22) samples.

Somatic SNVs Identified using GATK-MuTect2 (HS980 p22 vs HS980 (p38))
Combined Non-redundant SNVs (replicate-1, 2 & 3)
ANNOVAR annotation of SNVs
SNVs with Missence, Frameshift and Silent mutation annotation
Average Expression (RPKM) of gene from single-cell experiment (hES cells)

Chr	Start	End	Ref	Alt	dbSNP_rs-ID	Gene-ID	Annotation	Amino Acid Change	Avg. Expression (hESc)
chr1	17086940	17086941	AC	-	rs748266310	MST1L	Frameshift Deletion	c.383_384del:p.128_128del	0.80
chr1	229738614	229738615	AA	-	.	TAF5L	Frameshift Deletion	c.299_300del:p.100_100del	1.30
chr8	143958453	143958453	-	GGAA	.	CYP11B1	Frameshift Insertion	c.580_581insTTCC:p.H194fs	0.00
chr1	145349584	145349584	T	A	rs370191373	NBPF10	Non-synonymous	c.T6999A:p.D2333E	0.03
chr11	1018012	1018012	T	G	rs10751676	MUC6	Non-synonymous	c.A4789C:p.K1597Q	0.00
chr14	105417313	105417313	T	C	rs80275639	AHNAK2	Non-synonymous	c.A4475G:p.K1492R	0.98
chr15	82635194	82635194	T	C	rs1610794	GOLGA6L10	Non-synonymous	c.A1376G:p.E459G	0.00
chr18	14542791	14542791	C	T	rs201849570	POTEC	Non-synonymous	c.G355A:p.A119T	0.00
chr19	4511746	4511746	A	T	rs62115192	PLIN4	Non-synonymous	c.T2184A:p.N728K	0.16
chr19	54745682	54745682	C	T	rs111666280	LILRA6	Non-synonymous	c.G419A:p.R140Q	0.00
chr2	113147370	113147370	G	A	rs202082997	RGPD5,RGPD8	Non-synonymous	c.C3152T:p.T1051I	0.00
chr20	30231249	30231249	G	C	.	COX4I2	Non-synonymous	c.G290C:p.R97P	0.72
chr21	45994014	45994014	C	T	rs13051603	KRTAP10-4	Non-synonymous	c.C379T:p.P127S	0.00
chr5	79747478	79747478	C	A	.	ZFYVE16	Non-synonymous	c.C3557A:p.A1186E	7.01
chr6	17602910	17602910	G	A	rs144013791	FAM8A1	Non-synonymous	c.G802A:p.V268I	2.11
chr6	32549402	32549402	C	T	rs3205588	HLA-DRB1	Non-synonymous	c.G584A:p.R195Q	0.00
chr7	75124676	75124676	G	A	rs28422159	SPDYE5	Non-synonymous	c.G242A:p.G81D	0.11
chr7	100645807	100645807	G	A	rs112404953	MUC12	Non-synonymous	c.G11963A:p.G3988D	0.14
chr9	43915893	43915893	G	C	rs200215881	CNTNAP3B	Non-synonymous	c.G3741C:p.M1247I	0.15
chr11	1643096	1643096	G	A	rs59587741	KRTAP5-4	Synonymous	c.C228T:p.G76G	0.00
chr11	62296299	62296299	G	A	.	AHNAK	Synonymous	c.C5590T:p.L1864L	0.98
chr11	117077034	117077034	A	G	rs28590104	PCSK7	Synonymous	c.T2037C:p.T679T	6.60
chr12	125397475	125397475	C	A	rs11537760	UBC	Synonymous	c.G843T:p.G281G	134.65
chr18	14542867	14542867	C	T	rs200779556	POTEC	Synonymous	c.G279A:p.T93T	0.00
chr19	1881263	1881263	G	A	.	ABHD17A	Synonymous	c.C303T:p.C101C	0.44
chr2	234358746	234358746	C	T	rs754569132	DGKD	Synonymous	c.C2007T:p.F669F	1.90
chr20	60904081	60904081	G	A	rs544096167	LAMA5	Synonymous	c.C4266T:p.N1422N	4.91
chr3	195452689	195452689	C	T	rs141077164	MUC20	Synonymous	c.C702T:p.D234D	0.00
chr3	195508986	195508986	G	A	rs2948679	MUC4	Synonymous	c.C9465T:p.V3155V	0.00
chr6	31964664	31964664	G	A	rs12526327	C4A,C4B	Synonymous	c.G3735A:p.P1245P	0.00
chr12	40880507	40880507	A	G	.	MUC19	Unknown	UNKNOWN	0.02
chr7	26246132	26246132	T	G	.	CBX3	Splicing	exon3:c.167+2T>G	84.05

Highlighted in grey: genes with some clinical relevance reported in COSMIC/ClinVar

Supplemental Table S7. Clinical significance analysis of 11 normal participants from personal genome project UK.

Sample-ID	A	B	C	D	E	F	G	H
HS980 (p22) common to three replicates	4355521	2.05	20	20	2160	35/34	20/20	18/18
01_uk35C650	4105831	2.07	28	27	2102	33/32	30/29	15/15
02_uk2E2AAE	4133496	2.07	24	24	2039	31/31	19/19	18/18
03_uk2DF242	4128205	2.06	23	21	2026	31/31	21/21	21/20
04_uk740176	4248904	2.07	31	30	2084	29/28	18/17	12/11
05_uk33D02F	4188095	2.07	24	22	2109	25/25	17/16	12/12
06_uk0C72FF	4112224	2.07	24	24	2069	40/39	28/28	18/17
07_uk1097F9	4137176	2.07	18	16	2150	30/29	18/17	11/10
08_uk174659	4175392	2.06	16	14	2067	46/45	29/29	24/24
09_uk85AA3B	4179265	2.06	25	23	2049	28/28	21/21	15/15
10_uk481F67	4901623	2.07	28	28	1794	44/42	27/26	14/13
11_uk4CA868	3897315	2.07	23	22	2072	38/37	24/23	21/20

A: Total Germline SNVs
B: Transitions/Transversions (ts/tv)
C: SNVs with clinical significance (CLNID) as “Pathogenic” in ClinVar-20171029 database release
D: ClinVar annotated “Pathogenic” SNVs reported as COMMON in dbSNP (minor allele frequency (MAF) of >= 0.01 in at least one 1000 Genomes Phase III major population with at least two individuals from different families having the same minor allele)
E: SNVs with FATHMM prediction as “Pathogenic” in COSMIC (COSMIC Coding v-83) database
F: COSMIC annotated “Pathogenic” SNVs within COSMIC Cancer Gene Census / SNVs COMMON in dbSNP
G: COSMIC annotated “Pathogenic” SNVs within Bailey MH et al. Cancer Driver Genes / SNVs COMMON in dbSNP
H: COSMIC annotated “Pathogenic” SNVs within Shibata Cancer Driver Genes / SNVs COMMON in dbSNP
